# A Nonsense Mutation in Mouse *Tardbp* Affects TDP43 Alternative Splicing Activity and Causes Limb-Clasping and Body Tone Defects

**DOI:** 10.1371/journal.pone.0085962

**Published:** 2014-01-21

**Authors:** Thomas Ricketts, Philip McGoldrick, Pietro Fratta, Hugo M. de Oliveira, Rosie Kent, Vinaya Phatak, Sebastian Brandner, Gonzalo Blanco, Linda Greensmith, Abraham Acevedo-Arozena, Elizabeth M. C. Fisher

**Affiliations:** 1 MRC Mammalian Genetics Unit, Harwell, Oxfordshire, United Kingdom; 2 Department of Neurodegenerative Diseases and MRC Centre for Neuromuscular Diseases, UCL Institute of Neurology, London, United Kingdom; 3 Sobell Department of Motor Neuroscience and Movement Disorders and MRC Centre for Neuromuscular Diseases, UCL Institute of Neurology, London, United Kingdom; 4 Biology Department, University of York, York, United Kingdom; Mayo Clinic, United States of America

## Abstract

Mutations in *TARDBP*, encoding Tar DNA binding protein-43 (TDP43), cause amyotrophic lateral sclerosis (ALS) and frontotemporal dementia (FTD). Attempts to model TDP43 dysfunction in mice have used knockouts or transgenic overexpressors, which have revealed the difficulties of manipulating TDP43, whose level is tightly controlled by auto-regulation. In a complementary approach, to create useful mouse models for the dissection of TDP43 function and pathology, we have identified a nonsense mutation in the endogenous mouse *Tardbp* gene through screening an *N*-ethyl-*N*-nitrosourea (ENU) mutant mouse archive. The mutation is predicted to cause a Q101X truncation in TDP43. We have characterised *Tardbp^Q101X^* mice to investigate this mutation in perturbing TDP43 biology at endogenous expression levels. We found the *Tardbp^Q101X^* mutation is homozygous embryonic lethal, highlighting the importance of TDP43 in early development. Heterozygotes (*Tardbp^+/Q101X^*) have abnormal levels of mutant transcript, but we find no evidence of the truncated protein and mice have similar full-length TDP43 protein levels as wildtype littermates. Nevertheless, *Tardbp^+/Q101X^* mice have abnormal alternative splicing of downstream gene targets, and limb-clasp and body tone phenotypes. Thus the nonsense mutation in *Tardbp* causes a mild loss-of-function phenotype and behavioural assessment suggests underlying neurological abnormalities. Due to the role of TDP43 in ALS, we investigated potential interactions with another known causative gene, mutant superoxide dismutase 1 (*SOD1*). *Tardbp^+/Q101X^* mice were crossed with the *SOD1^G93Adl^* transgenic mouse model of ALS. Behavioural and physiological assessment did not reveal modifying effects on the progression of ALS-like symptoms in the double mutant progeny from this cross. In summary, the *Tardbp^Q101X^* mutant mice are a useful tool for the dissection of TDP43 protein regulation, effects on splicing, embryonic development and neuromuscular phenotypes. These mice are freely available to the community.

## Introduction

Amyotrophic lateral sclerosis is a progressive neurodegenerative disease characterised by the degeneration of upper and lower motor neurons, resulting in denervation and atrophy of skeletal muscles, leading to paralysis. Disease course is rapid, with death typically occurring within 3 to 5 years of diagnosis, usually due to paralysis and respiratory insufficiency [1]. Although the majority of ALS cases occur sporadically, approximately 10% of cases are familial (fALS) and our understanding of the genetic causes has expanded dramatically over the past few years [2], [3].

Genetic and pathological findings suggest ALS lies within a spectrum of diseases including frontotemporal dementia, a neurodegenerative disorder characterised by degeneration and atrophy of specific cortical areas [1], [3]. The association between ALS and FTD was strengthened following identification of TDP43, a nuclear RNA-binding protein, as a major constituent of ubiquitinated cytoplasmic aggregates in degenerating neurons in *post mortem* tissue from both sporadic and fALS and FTD [4]–[6]. Subsequently, mutations in *TARDBP,* the gene encoding TDP43, were identified in ALS and FTD [7]–[11]. Mutations in *TARDBP* account for approximately 4% of fALS, a smaller proportion than those caused by mutations in *SOD1* (10–20%) or the repeat expansion in *C9orf72* (40%), although these figures vary within different populations [12]–[17]. However, the observation of TDP43 pathology in both sporadic ALS and *C9orf72* patients [5], and other neurodegenerative disorders [18], [19], suggests that TDP43 dysfunction may be widely relevant to ALS pathogenesis and neurodegeneration generally. TDP43 pathology is not observed in *SOD1*-ALS, which has led to the suggestion that divergent disease processes may underlie these forms of ALS. Currently there is evidence for and against an interaction between TDP43 and SOD1 [20]–[24].

TDP43 is a nuclear protein that plays crucial roles in gene expression and alternative splicing [25], [26], embryogenesis [27]–[30], the stress response [31]–[36], neuronal functions such as neurite outgrowth [37]–[39] and many other cellular processes [30], [40], [41]. It is currently unknown whether mutant TDP43 causes neuronal degeneration through loss of one of these functions or via a novel gain-of-function mechanism, or through both [1], [42], [43].

To investigate the toxic effect of mutant TDP43, both overexpressing and knockout rodent models have been developed [44]–[46]. These models show overexpression of wildtype or mutant TDP43 is dose-dependently toxic and results in early mortality, recapitulating some FTD- and ALS-like phenotypes and pathological features. To address whether mutant TDP43 acts through a loss-of-function mechanism, knockout models have been developed by genetically disrupting mouse *Tardbp* [27]–[29]. Homozygous *Tardbp* null animals all die between embryonic day (E) 3.5 and 8.5 [27]–[29]. In an attempt to avoid the developmental lethality of TDP43 deficiency, a mouse with a tamoxifen-inducible null allele of *Tardbp* has also been produced [47]. However, disruption of both *Tardbp* alleles in adult mice led to rapid mortality within nine days, associated with drastic metabolic alterations [47].

Ablation of one allele of *Tardbp* does not affect TDP43 protein levels, leading to the finding that TDP43 autoregulates its protein levels *in vivo* [25], [27]–[29], [48], [49]. Disruption of autoregulation is a potential pathomechanism of mutant TDP43 [50], [51]. Intriguingly, despite the absence of overt pathology or perturbation of TDP43 protein levels, one study noted impaired motor performance in aged *Tardbp^+/−^* mice [28].

Although the severe effects of systemic TDP43 deficiency are a barrier to investigating loss-of-function, conditional deletion of *Tardbp* from motor neurons (using loxP flanked *Tardbp* mice crossed to Hb9-Cre recombinase expressing animals) resulted in delayed weight-gain, impaired rotarod performance, loss of spinal cord motor neurons and ALS-like pathology [52], [53]. Similarly, selective disruption of *Tardbp* in cells (including lower motor neurons) expressing the vesicular acetylcholine transporter, led to progressive decreases in bodyweight and rotarod performance, albeit with no effect on lifespan [53]. Progressive motor impairments in these mice were associated with denervation and atrophy of skeletal muscles, but not loss of spinal cord motor neurons [53].

Loss of TDP43 function in rodents can have widespread effects on alternative splicing. Striatal depletion of TDP43 in adult mice, using antisense oligonucleotides, alters the cassette exon inclusion/exclusion of gene targets, many of which are associated with neurodegeneration [25]. Furthermore, in transgenic mice which overexpress mutant TDP43, alternative splicing patterns of target genes suggests both a loss- and gain-of-function effects [54]. Although transgenic rodents overexpressing human wildtype or mutant TDP43 model some features of ALS and FTD, and TDP43 dysfunction, it is important to remember that autoregulation of TDP43 can cause downregulation of endogenous mouse TDP43 [55], and leading to potential loss-of-function effects.

Given the dose-dependent toxicity of TDP43 in transgenic and knockout rodents, we screened an ENU archive to identify point mutations in the mouse *Tardbp* gene. ENU is a powerful mutagen that creates heritable point mutations throughout the genome [56]–[61]. Male mice are injected with ENU, which ultimately produces a unique array of random point mutations in their sperm, in a dose-dependent manner [62]. Injected mice are mated and sperm from their male offspring harvested, with matching DNA samples, which can be screened for mutations. From screening the ENU archive at the Medical Research Council Mammalian Genetics Unit (Harwell, UK) [56], we identified a mouse *Tardbp* mutation predicted to cause a Q101X truncation in the N-terminus of TDP43.

The new TDP43 Q101X strain was assessed for TDP43 expression and function, and mouse survival, behaviour and motor function. We also carried out a cross to transgenic mice carrying a mutant *SOD1* gene, which models ALS, to determine if there were any interaction effects between the two mutant genes. We found the *Tardbp^Q101X^* mutation affected alternative splicing of selected target genes and caused behavioural phenotypes, in the absence of motor dysfunction or deleterious effects of mouse survival. Progeny of the novel cross to *SOD1^G93Adl^* transgenic mice did not reveal interaction effects between the novel mutation in *Tardbp* and the *SOD1^G93A^* transgene.

## Methods

### Ethics Statement

Mice were bred and maintained by MRC Harwell and UCL Institute of Neurology. Experiments were performed under licence from the UK Home Office and following approval from the local ethical review panels: Ethical Review Panel of MRC Harwell and Ethical Review Panel of the MRC Prion Unit, UCL Institute of Neurology. Surgery was performed under terminal anaesthesia and all efforts were made to minimise suffering.

### Identification of an ENU-induced Mutation in *Tardbp*

DNA from the MRC Harwell ENU archive (http://www.har.mrc.ac.uk/services/dna_archive/) was screened with the LightScanner platform (Idaho Technology Inc., USA). All exons of *Tardbp* were screened in DNA from ∼10,000 F1 ENU mutagenised animals and potential mutations confirmed with Sanger sequencing (GATC, Germany). This led to the identification of a C to T base mutation within exon 3 which is predicted to cause a Q101X nonsense (glutamine to stop codon) change in TDP43 protein.

### Genetic Background

Male C57BL/6J mice treated with ENU and were crossed to C3H/HeH females. F1 progeny (C3H/HeH.C57BL/6J) were rederived and male F1 animals had sperm and DNA samples taken for archiving. F1 DNA was screened for mutations in *Tardbp*, and the *Tardbp^Q101X^* strain was rederived from F1 sperm, used for in vitro fertilisation of C57BL/6J oocytes. All mice used for phenotyping had been back-crossed 3–4 generations onto a C57BL/6J background. Third generation (N3) back-cross C57BL/6J females were crossed with congenic C57BL/6J *SOD1^G93Adl^* male mice [63] to generate wildtype, single and double mutant progeny.

### Genotyping

The *Tardbp* Q101X allele was genotyped using a Qiagen pyro-sequencer. Extracted DNA (5 µg/µl) was amplified and sequenced following manufacturer’s instructions at 55°C annealing temperature. Primers used were forward: CAAAAGGAAAATGGATGAGAC, reverse: AGTTGTTTTCCAGGGGAGAC, sequencing forward: GAAAGTGAAAAGAGCAGTC, which flank the site of mutation and bind witin exon 3. The pyro-sequencer converts luciferin to oxyluciferin to produce light resulting in a quantitative read-out. Thus, to measure the relative abundance of the Q101X *Tardbp* transcript compared to the wild-type transcript, cDNA was generated from brain and analysed on the pyro-sequencer with the quantitative readout for the oxyluciferin generated from the wildtype (C) and mutant (T) bases used to determine relative levels of transcripts containing each base.

*SOD1^G93Adl^* mice were maintained as hemizygotes and genotyped with transgene and control specific primers, as described on the Jackson Laboratory Mice Database (http://jaxmice.jax.org/strain/002300). Transgene primers, amplifying across exon 4: forward: CATCAGCCCTAATCCATCTGA, which binds within intron 3, and reverse: CGCGACTAACAATCAAAGTGA, which binds within intron 4. In order to control for the reaction, the mouse gene *Scna* was also amplified as the control gene to validate the mouse DNA sample. *Scna* primers, forward: ATCTGGTCCTTCTTGACAAAGC, reverse: AGAAGACCAAAGAGCAAGTGACA, which both bind within exon 4. These produced amplicons of 236 bp and 130 bp respectively, with presence or absence of the *SOD1^G93A^* fragment used to determine genotype.

### Molecular Analysis of TDP43 Alternative Splicing

RNA was extracted from tissue using TRIzol (Life Technologies, Paisley, UK) and cDNA was produced using Applied Biosystems cDNA generation kit (Life Technologies, Paisley, UK) following the manufacturer’s protocol. Oligo dT primers were used to generate the cDNA. To assess splicing reactions, 1 µl cDNA at ∼ 50 ng/µl was used for polymerase chain reaction (PCR) with a Qiagen master mix (201445; Qiagen, Netherlands) with 34 thermo cycles. The PCR product was electrophoresed on a 3% agarose gel and visualised using a Bio-Rad ChemiDoc (Bio-Rad, California, USA). The forward and reverse primers for each reaction were located in exons either side of the known alternatively spliced exon for each target gene. *Sort1*– forward (exon 17): CAGGAGACAAATGCCAAGGT, reverse (exon 19): TGGCCAGGATAATAGGGACA, *Pdp1* - forward (5′UTR): GTGCTGAGTGAGGGAAGGAC, reverse (exon 2): TGCAGTGCCATAGATTCTGC, *Kcnip2*– forward (exon 1): CGGCTCCTATGACCAGCTTA, reverse (exon 4): GGAGTTGTTCCAGACCCTCA, *Kcnd3*– forward (exon 4): GGCAAGACCACCTCACTCAT, reverse (exon 6): AGTGGCTGGACAGAGAAGGA, *Dnajc5*– forward (exon 3): CTCTATGTGGCGGAGCAGTT, reverse (exon 5): GCTGTATGACGATCGGTGTG. Real time PCR (rtPCR) was carried out with fast SYBR Green using an ABI7500 machine. ΔΔ_CT_ was calculated with the control gene *S16* to quantify normalized *Tardbp* levels. *Tardbp* was amplified with exon 2 forward: GGAATCCCGTGTCTCAGTGT with exon 3 reverse: AGGAAGCATCTGTCTCATCCA and exon 3 forward: CTCCCCTGGAAAACAACTGA with exon 4 reverse: AAAGCCAAACCCTTTCGAGT.

### Assessment of TDP43 Protein Levels

Protein was harvested from tissue and cells by homogenisation in RIPA buffer with protease inhibitor cocktail (Roche). Centrifugation (13,200×g at 4°C for 30 minutes) was used to purify the soluble fraction. SDS-page gels were Invitrogen Novex BisTris 10% gels (Life Technologies, Paisley, UK) and transferred onto immobilon low fluorescence PVDF membrane (Millipore, Massachusetts, USA). Membranes were blocked in PBS with 0.2% tween and 5% milk. Anti TDP43 antibodies were from: ProteinTech (12892-1-AP) and N-terminus antibodies from Cosmo Bio (CAC-TIP-TD-P07, Cosmo Bio, California, USA) and Abcam (ab50930, Abcam, Cambridge, UK). Control actin was labelled using Abcam primary (AC-15). Visualisation with the Li-Cor Odyssey system (LI-COR Biosciences, Nebraska, USA) used mouse and rabbit specific red and green fluorescently conjugated secondary antibodies (Li-Cor IRDye 680RD, 926-32220/800CW, 926-32211).

### Embryonic Lethality of *Tardbp^Q101X/Q101X^* Mutant Mice

Embryonic lethality was assessed by culling female mice at selected days post conception using UK Home Office defined Schedule 1 methods. Embryos were harvested and tissue collected for genotyping.

### Lifespan

All mice were maintained and assessed in accordance with UK Home Office project licence regulations. Mice were weighed every two weeks until the endpoint of the assessment was reached, or until a humane endpoint, defined as when mice showed either hunched posture, 20% bodyweight loss or limb paralysis. Only females were used for the lifespan analysis of *Tardbp^Q101X^* mice, as aged males were used for pathological analysis at scheduled time points.

### Behavioural Analysis of Mice

Grip-strength was longitudinally assessed (BioSeb, France) by measurements using all four paws and the two front paws alone. Recordings were taken the week following the SHIRPA assessment, from 8 weeks of age to 104 weeks of age.

Motor performance on an accelerating rotarod was assessed with three trials per day, on three days within the testing week. The average value of all nine runs was recorded (Ugo Basile, Varese, Italy). The rotarod accelerated from 4 to 40 revolutions per minute over a maximum of 300 seconds. Rotarod was assessed from 8 weeks of age to 56 weeks of age and was measured in the same week as the grip strength test.

Behavioural analysis of mice was performed using the SHIRPA (SmithKline Beecham, Harwell, Imperial College, Royal London Hospital, phenotype assessment) protocol as previously described [64]. Phenotyping was carried out blind to mouse genotype. SHIRPA was undertaken every two months from 7 weeks of age for the *Tardbp^+/Q101X^* phenotyping cohort; female *Tardbp* ^+/+^ n = 12, female *Tardbp^+/Q101X^* n = 15, male *Tardbp* ^+/+^n = 16, male *Tardbp^+/Q101X^* n = 16. Beyond one year, only the female cohort was phenotyped up to end stage or two years of age. For the double mutant cross between *Tardbp^+/Q101X^* and *SOD1^G93Adl^*, progeny were phenotyped monthly from 7 weeks of age up to endstage between 31–38 weeks. This cohort contained, females: *Tardbp* ^+/+^ n = 10, *Tardbp^+/Q101X^* n = 5, *Tardbp* ^+/+^, *SOD1^G93Adl^* n = 14, *Tardbp^+/Q101X^*, *SOD1^G93Adl^* n = 14. Males: *Tardbp* ^+/+^ n = 12, *Tardbp^+/Q101X^* n = 10, *Tardbp* ^+/+^, *SOD1^G93Adl^* n = 15, *Tardbp^+/Q101X^*, *SOD1^G93Adl^* n = 14.

Within the SHIRPA, limb-clasping was assessed by suspending the mice by the tail, with a score of 0 given for no clasping and 1 given for front or hind-paw clasping. For analysis, only mice which showed clasping over two successive assessments and maintained this phenotype in all remaining assessments were scored as limb-clasping. Body tone was assessed by holding mice near the base of the tail; front paws were placed to grip a grid above the SHIRPA arena and the hindlimbs were suspended slightly above the grid. With the free hand, the thumb and index finger were placed around the pelvic region and lower thorax and then rounds of lateral compression, while continuously keeping the thumb and finger against the mouse, were used to feel muscular tone with a reflexive elicited response. If mice were anxious and elicited a jerk/jump response they were then re-tested. Mice were scored 2 for normal body tone, 1 for soft tone, and 0 for flaccid tone. Mice were only considered to have body tone phenotype if they showed flaccid tone over two consecutive assessments and maintained this phenotype.

Startle response and pre-pulse inhibition were tested as described on the Empress database (http://empress.har.mrc.ac.uk). The *Tardbp^+/Q101X^* cohort was assessed at 10 and 22 weeks of age and the *Tardbp^+/Q101X^*, *SOD1^G93Adl^* cohort assessed at 10, 18 and 22 weeks of age.

The Open Field test was undertaken to assess both motor- and anxiety-based behaviour, and analysed with software from Noldus (Wageningen, Netherlands). The test was completed as described on the Empress database but was modified to increase the recording time from 20 to 30 minutes. The *Tardbp^+/Q101X^* cohort was assessed at 14 and 30 weeks of age. Progeny from the *Tardbp^+/Q101X,^ SOD1^G93Adl^* cross were assessed at 14 weeks of age.

### Pathology

Brains from *Tardbp^+/+^* and *Tardbp* ^+/Q101X^ male mice (both n = 3) were harvested for histopathological analysis at one year of age. Mice were perfused transcardially with 0.9% saline followed by 4% paraformaldehyde (PFA). Following dissection, brains were transferred to formalin and subsequently embedded in paraffin wax. Sections were stained with haematoxylin and eosin, and antibodies against GFAP (3 ug/mL, DAKO, Z0334), IBA-1 (1.7 ug/mL, WAKO Chemicals, 019-19741), p62 (5.6 ug/mL, Progen Biotechnik, GP62-C), MBP (2 ug/mL, Covance, SMI-94R) and neurofilament (0.5 ug/mL, Sigma, N5389) using a Ventana automated immunohistochemical staining machine (Ventana Medical Systems, Tuscon, USA). Once incubated with appropriate secondary antibodies, immunoreactivity was developed with 3,30-diaminobenzidine and counterstained with haematoxylin.

### Primary Embryonic Motor Neuron Culture and Quantification of Neurite Outgrowth

Mixed ventral horn cultures were prepared as previously described [65]. Briefly, primary embryonic motor neurons were isolated on E13.5, following cervical dislocation of pregnant females and hysterectomy. Ventral horns were dissected from individual embryos and dissociated by 10 minute incubation with trypsin (final concentration 0.025%, Type XII-S, Sigma Aldrich, Paisley, UK) and three trituration steps in 400 µl L-15, 50 µl 4% bovine serum albumin (BSA, Sigma Aldrich,) in L-15 and 50 µl DNase (1 µg/ml, Sigma Aldrich,). 1 ml 4% BSA was added to the cell suspension to form a cushion and cells were centrifuged at 239xg, room temperature for 5 minutes. Cell pellets were resuspended and then plated onto glass coverslips (precoated with polyornithine and laminin for at least 2 hours each) and maintained in neurobasal medium, supplemented with 1% penicillin-streptomycin (50 units/ml penicillin, 50 µg/ml streptomycin), 2% B27 supplement, 2% horse serum, 0.05% 50 mM β-mercaptoethanol, 0.5 mM L-glutamine (all from Invitrogen, Paisley, UK), 0.1 ng/ml brain-derived neurotrophic factor, 0.1 ng/ml glial-derived neurotrophic factor and 0.5 ng/ml ciliary neurotrophic factor (all from Peprotech, London, UK).

After 18 hours *in vitro*, coverslips were immunostained with β-III-tubulin (Covance, Harrogate, UK) to identify neuronal cells and fluorescent images were captured at×20 magnification with a Leica DFC colour camera (Leica, Wetzlar, Germany) running Leica Application Suite Version 2.8.1 (Leica, Wetzlar, Germany). From 3 independent cultures, 10 random fields were imaged from 3 coverslips per genotype. To assess neurite outgrowth in primary embryonic motor neuron cultures, only cells identified with β-III-tubulin, with at least 3 neurites whose full length could be seen, were selected. The length of every selected neurite and branch was measured with Metamorph (Molecular Devices, Berkshire, UK) and data transferred into Microsoft Excel (Microsoft Corporation, Redmond, Virginia, US), where mutant values were expressed as a percentage of wildtype values. Data were plotted for the mean neurite length, and the mean longest neurite length.

### *In vivo* Assessment of Neuromuscular Function

Neuromuscular function was assessed *in vivo* in anaesthetised mice (4.5% chloral hydrate, i.p, 1 ml/100g bodyweight; Sigma Aldrich), as previously described [66], [67]. Anaesthesia was checked regularly throughout the procedure and supplemented when necessary with a quarter of the initial dose of anaesthetic.

The distal tendons of the tibialis anterior (TA) and extensor digitorum longus (EDL) muscles were exposed, cut and attached to isometric force transducers (Dynamometer UFI Devices, Welwyn Garden City, UK), which were connected to a Multitrace Recorder (Lectromed Multitrace 2, Letchworth Garden City, UK), and a Picoscope recorder (Pico Technology, Cambridgeshire, UK) which captured the data.

The sciatic nerve was exposed in the mid-thigh region and sectioned proximally before being placed over a platinum stimulating electrode and kept moist with 0.9% saline. Muscle length was adjusted for maximum twitch force. The sciatic nerve was then stimulated with 0.02 ms square wave pulses at a supramaximal voltage (10V) to record maximum single twitch force. Maximum tetanic force was determined by stimulating the sciatic nerve with trains of stimuli at 40, 80 and 100 Hz for 450 ms. From the maximum twitch force recorded, muscle contraction characteristics were determined by measurement of the time taken to reach peak contraction (time-to-peak; TTP) and the time to reach half relaxation (½RT). Muscle fatigue characteristics of the EDL were assessed by a fatigue test, in which the muscle was stimulated at 40 Hz for 250 ms per second, for 180 seconds, and the resulting muscle contractions recorded on a pen electrode (Lectromed Multitrace 2). The fatigue index (FI), a measure of fatiguability, was determined from the trace by expressing the final force (T_180_) as a ratio of initial force (T_0_).

To determine the number of surviving motor units innervating the EDL, the sciatic nerve was stimulated with voltage of increasing intensity, from a sub-threshold voltage to maximum, resulting in the gradual recruitment of motor units, reflected as stepwise increments in twitch tension, which were counted to estimate the number of functional motor units.

### Morphological Assessment of Motor Neuron Survival

Following the acute physiological experiment, the mice were terminally anaesthetised with an overdose of 4.5% chloral hydrate (i.p.) and transcardially perfused with 0.9% saline followed with 4% PFA. Spinal cords were dissected from the spinal column and left overnight in 4% PFA at 4°C before cryoprotection in 30% sucrose in PBS at 4°C. Cross-sections of the lumbar region (L2-L6) of the spinal cord were cut at 20 µm thickness on a cryostat and Nissl stained with gallocyanin [68]. Motor neuron survival was established by counting the number of large polygonal neurons with a minimum diameter of 20 µm within the sciatic motor pool [69]. Only those cells with a clear nucleolus and distinct Nissl-dense cytoplasm were counted in every 3rd section to prevent recounting the same neurons twice in consecutive sections. Counting was performed blind to genotype using a Leica DMR microscope and using a minimum for 40 sections.

### Neuromuscular Junction Immunofluorescence

Mice were killed by cervical dislocation. Abdominal muscles were dissected, isolated in PBS, stained for 10–20 minutes in TRITC-conjugated α-bungarotoxin (Biotium, Inc.), washed in PBS and fixed for 30–60 minutes in 4% paraformaldehyde. Axons were visualised by staining with anti-150 kDa Neurofilament M rabbit and SV2 mouse antibodies overnight at 4°C at dilutions of 1∶250 and 1∶200, respectively. After washing for 1 hour in PBS, samples were incubated with secondary antibody (150 kDa Neurofilament M: goat anti-rabbit IgG FITC-conjugated antibody, 1∶100 dilution; SV2: goat anti-mouse IgG FITC-conjugated antibody, 1∶100 dilution) and subsequently washed with PBS. Whole muscles were mounted in Mowiol and imaged using a BioRad Radiance 2000 confocal microscope mounted on a Nikon Eclipse E600FN platform, using 20x and 40x water immersion objectives.

### Statistical Analysis

Kaplan-Meier Log rank statistic was used to compare phenotypes in the SHIRPA to define age of phenotype onset. For other tests including grip strength, rotarod, startle response, open field, RNA and protein levels; comparisons were made using a two-tailed ANOVA test with Least Squared Difference (LSD) and/or Bonferroni post-hoc tests or two tailed T-test. Mean neurite length and axon length were analysed using a multi-level model in Stata 12 (StataCorp, Texas, USA), to compare neurite and axon lengths between wildtype and mutant motor neurons from independent experiments. For *in vivo* assessment of neuromuscular function a Mann-Whitney U-test was used to compare between *Tardbp^+/+^* and *Tardbp^+/Q101X^* mice at 18 months of age. A one-way ANOVA, with Tukey’s test was used to compare groups at 32–33 weeks of age.

## Results

### Identification of an ENU-induced Point Mutation in Mouse *Tardbp*

**Figure 1:**
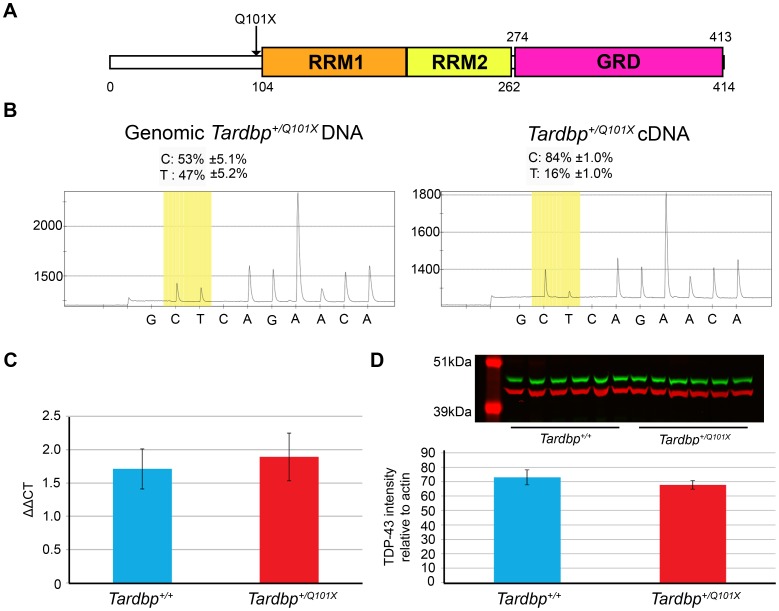
*Tardbp^+/Q101X^* mice have reduced mutant transcript but normal TDP43 protein levels. (**A**) Location of the predicted Q101X truncation in TDP43. All coding exons of *Tardbp* were screened for mutations using denaturing high performance liquid chromatography, with a C to T mutation identified in exon 3 which is predicted to encode a Q101X truncation, occurring before the RNA recognition motifs (RRM1/2) and the glycine-rich domain (GRD). Numbers denote amino acids in the full-length 414 residue protein. (**B**) Genomic DNA and transcript levels were assessed in brain tissue from male *Tardbp^+/+^* (n = 2) and *Tardbp^+/Q101X^* (n = 5) mice at 18 months of age. Data are mean±standard deviation. (Left panel) Genomic DNA: in *Tardbp^+/Q101X^* mice, the wildtype and mutant alleles in genomic DNA are detected at a ratio of ∼1∶1. (Right panel) cDNA: levels wildtype and mutant transcripts were quantified by pyro-sequencing; wildtype base is C and mutant base is T. RNA converted to cDNA shows a ratio of 5.25∶1 for wildtype:mutant levels. The pyro-sequencer measures the relative areas under each curve, allowing comparison of relative levels of the two transcripts p<0.001 with Fisher’s test. (**C**) *Tardbp* transcript levels, assessed using primers spanning exons 2 to 3 were not altered in brain tissue from male mice at 18 months of age. Mean ΔΔ_CT_ of *Tardbp^+/+^* = 1.7±0.3 (n = 5), *Tardbp^+/Q101X^* = 1.9±0.4 (n = 5), p = 0.71 (students two-tailed t-test). Data are mean±standard deviation. (**D**) No difference in full-length TDP43 protein levels (relative to loading control) between *Tardbp^+/+^* (73.0±5.2) and *Tardbp^+/Q101X^* (67.8±3.0) mice, using antibody directed against C-terminus of TDP43 (Proteintech 12892-1-AP). TDP43 levels (upper panel, green) were assessed from brain tissue with 5 male mice per genotype at 18 months of age. Actin (upper panel, red) was used as a loading control. Data are mean±SEM.

The MRC Harwell ENU archive of over 10,000 DNAs was screened and a C to T mutation in exon 3 of *Tardbp* was identified. The mouse strain was rederived onto a C57BL/6J background using standard *in vitro* fertilisation techniques. The mutation is predicted to cause a Q101X truncation in the N-terminus of TDP43 whose longest isoform in mouse is 414 amino acids. The truncation occurs before any predicted functional domains of the protein (**Figure 1A**).

### The Q101X Mutation in *Tardbp* is Homozygous Embryonic Lethal

To investigate embryonic lethality, heterozygous (*Tardbp^+/Q101X^*) mice were intercrossed to produce homozygous animals. No *Tardbp^Q101X/Q101X^* progeny were detected at birth, or between E7.5-11.5 (n = 47 embryos) or earlier at E6.5 (n = 25 embryos; **Table 1**). Therefore the *Tardbp* ^Q101X/Q101X^ mice die in early development prior to E6.5.

**Table 1:** *Tardbp^Q101X/Q101X^* embryos die in development before E6.5.

		Genotype			
Timed mating	n	*Tardbp* ^+/+^	*Tardbp* *^+/Q101X^*	*Tardbp* ^Q101X/Q101X^	Resorptions
E7.5–11.5	47	12	21	0	14
E6.5	25	9	11	0	5

Timed matings were set-up to establish survival of *Tardbp* ^Q101X/Q101X^ mice. No homozygous embryos were identified at time-points assessed. We were unable to genotype resorptions. At E6.5 χ2 = 8.3, p = 0.0158 versus an expected ratio of 1∶2:1– *Tardbp* ^+/+^: *Tardbp^+/Q101X^*: *Tardbp* ^Q101X/Q101X^.

### Reduced Level of Mutant Transcript but Normal Level of Wildtype Transcript in *Tardbp^+/Q101X^* Brain

As expected, genomic DNA from *Tardbp^+/Q101X^* mice showed equal levels of wildtype (53.0%±5.2%) and mutant alleles (47.0%±5.2%) (**Figure 1B**). We then investigated whether the Q101X mutation had effects on *Tardbp* transcript abundance. Using a Qiagen pyro-sequencer with primers flanking the mutation, we assessed transcript levels from brain cDNA in *Tardbp^+/Q101X^* mice and found a significant difference in the relative abundance of wildtype to mutant transcripts at 18 months of age (84%±1%: 16%±1%, p<0.001; **Figure 1B**) and 3 months of age (86%±1%: 14%±1%, p<0.001) – A ratio of 5.25∶1 instead of the anticipated 1∶1 ratio. Using this assay we can assess whether transcripts are wildtype or mutant, but we cannot distinguish different wildtype transcripts, however it demonstrates a greatly reduced level of mutant compared to wildtype transcript in *Tardbp^+/Q101X^* mice.

We went on to assess transcript levels by rtPCR. As the *Tardbp^Q101X^* mutation occurs within exon 3, we quantified *Tardbp* transcript levels using primers for regions spanning exons 2 to 3 and exons 3 to 4 (data not shown) in brain tissue from 18 month old mice. Both sets of primers amplify regions either side of the *Tardbp^Q101X^* mutation and do not overlap the mutation. Using these primers, transcript abundance did not differ between *Tardbp^+/+^* and *Tardbp^+/Q101X^* mice (1.7±0.3 versus 1.9±0.4 respectively; **Figure 1C**), demonstrating equivalent levels of wildtype transcripts.

Thus in heterozygous mouse brain (1) the mutant transcript is detectable, but at significantly lower levels than the wildtype transcript, and (2) levels of *Tardbp* transcripts including exon 2,3 and 4 are the same as in wildtype littermates.

### No Detectable Truncated but Normal Full-length TDP43 Protein Levels in *Tardbp^+/Q101X^* Mice

We assayed for truncated TDP43 Q101X protein by probing western blots with antibodies (Cosmo Bio: CAC-TIP-TD-P07, Abcam: ab50930) directed against the N-terminus of TDP43, and no truncated protein was detected in brain tissue from heterozygotes at 18 months of age (Figure S1A, B).

As previous reports have demonstrated that TDP43 autoregulates its protein levels [25], [28], [48], [49], and because transcript levels are the same in wildtype and mutant mouse brain, we assessed TDP43 protein levels using the Li-Cor Odyssey system and normalising to alpha actin content. Using an antibody directed against the C-terminus of TDP43 (ProteinTech: 12892-1-AP), a region beyond the predicted point of truncation, on brain from male mice at 18 months of age, no difference was seen in TDP43 protein levels (relative to loading control) between *Tardbp^+/+^* (73.0±5.2) and *Tardbp^+/Q101X^* (67.8±3.0) mice (n = 6 per genotype) (**Figure 1D**). Similar results were obtained with this antibody in spinal cords at 18 months of age for both soluble and insoluble fractions (**Figure S1C–D**) as well as at 3 or 12 months of age in brain or in spinal cord at 3 months, or in mouse embryonic fibroblasts (data not shown).

Thus *Tardbp^+/Q101X^* mice have equivalent, wildtype *Tardbp* transcript and TDP43 protein levels as wildtype littermates, but also have low levels of mutant transcripts, although no mutant protein that we could detect in brain tissue.

### Loss of Splicing Function of Selected Target Genes in *Tardbp^+/Q101X^* Mice

Although *Tardbp^+/Q101X^* mice showed overall normal wildtype TDP43 protein levels, we examined whether these mice retained complete TDP43 function. Depletion of TDP43, therefore a loss-of-function, has been previously shown to disrupt genome-wide cassette exon inclusion/exclusion in target genes [25]. Using splicing patterns of TDP43 target genes as a paradigm to assess TDP43 function, we characterised the exon inclusion/exclusion patterns of five established target genes: *Sort1*, *Pdp1*, *Dnajc5*, *Kcnd5* for which TDP43 promotes exon skipping, and *Kcnip2* for which TDP43 promotes exon inclusion.

**Figure 2:**
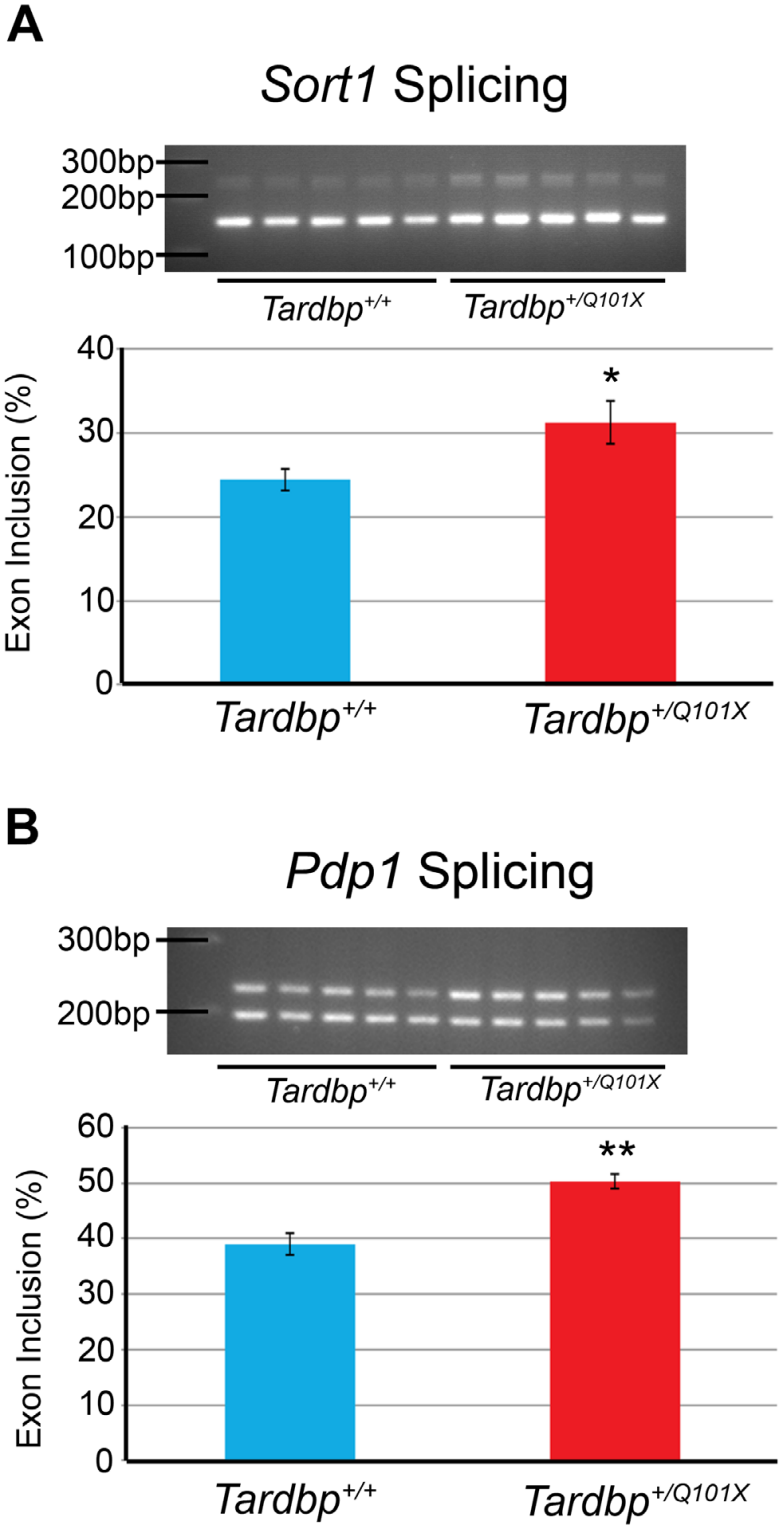
The Q101X mutation in *Tardbp* alters splicing of TDP43 targets *Sort1* and *Pdp1*. (**A**) Exon inclusion of *Sort1*, determined from cDNA generated from brain from 18-month old mice, is significantly increased in *Tardbp^+/Q101X^* (n = 5) mice compared to *Tardbp^+/+^* mice (n = 5). Bar charts show abundance of exon-included transcript relative to total transcript (included and excluded). *Sort1* exon inclusion: *Tardbp^+/+^*24.4%±1.3% versus *Tardbp^+/Q101X^* 31.3%±2.6%, p = 0.044 (student’s two-tailed t-test). Data are mean±SEM. (**B**) Exon inclusion of *Pdp1* is also significantly increased in brain tissue from *Tardbp^+/Q101X^* mice at 18 months of age. Exon inclusion: *Tardbp^+/+^*39.0%±2.0% (n = 5), *Tardbp^+/Q101X^* 50.3%±1.3% (n = 5), p = 0.0013 (student’s two-tailed t-test). Data are mean±SEM.

With RNA extracted from brain tissue at 18 months of age, we found significant differences in the splicing patterns of *Sort1* (exon 18 inclusion: *Tardbp^+/+^*24.4%±1.3%, *Tardbp^+/Q101X^* 31.3%±2.6%); **Figure 2A**) and *Pdp1* (exon 1 inclusion: *Tardbp^+/+^*39.0%±2.0%, *Tardbp^+/Q101X^* 50.3%±1.3%; **Figure 2B**) but no difference in the splicing patterns of *Dnajc5*, *Kcnd3* and *Kcnip2* (data not shown). This shows that in two target genes in the brain, the splicing function of TDP43 in *Tardbp^+/Q101X^* mice is altered in a manner similar to that following TDP-43 depletion [25], indicating possible partial loss-of-function. The altered splicing pattern for *Sort1* was also confirmed with RNA extracted from mouse embryonic fibroblasts (data not shown).

Loss of TDP43 affects neurite outgrowth in a neuroblastoma cell line, primary mouse cortical neurons and *Drosophila* neurons [37], [70], [71]. Following on from the potential loss of splicing function of at least some TDP43 target genes in the *Tardbp^+/Q101X^* mice, we assessed neuronal development in these animals by establishing primary embryonic motor neuron cultures and measuring neurite outgrowth after 18 hours *in vitro.* We observed no difference in mean longest neurite length (*Tardbp^+/Q101X^* = 93.2%±4.4% of *Tardbp^+/+^* value) or mean neurite length (*Tardbp^+/Q101X^* = 101.1%±11.2% n = 117 neurons, of *Tardbp^+/+^* value, n = 175 neurons) compared to *Tardbp^+/+^* motor neurons (**Figure S2**).

### Behavioural Characterisation of *Tardbp^+/Q101X^* Mice

**Figure 3:**
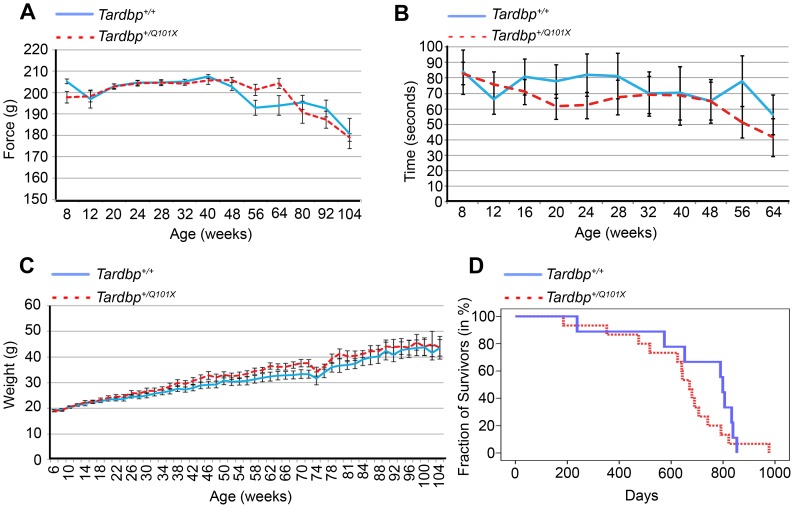
Grip-strength, locomotion, body weight, survival of *Tardbp^+/Q101X^* mice. (**A**) Grip-strength of female *Tardbp^+/Q101X^* mice is not significantly different from that of *Tardbp^+/+^* mice. Measurements represent average force (grams) in duplicate over all four limbs. Data are mean± SEM. *Tardbp^+/+^* n = 9, *Tardbp^+/Q101X^* n = 15. (**B**) Rotarod performance of female *Tardbp^+/Q101X^* mice is not significantly different from that of female *Tardbp^+/+^* mice. Measurements are time (seconds) on the accelerating rotarod averaged from 9 trials over 3 days. Data are mean± SEM. *Tardbp^+/+^* n = 13, *Tardbp^+/Q101X^* n = 15. (**C**) No significant differences in bodyweight of *Tardbp^+/+^* and *Tardbp^+/Q101X^* female mice. Bodyweight was recorded every two weeks. *Tardbp^+/+^* n = 9, *Tardbp^+/Q101X^* n = 15. Data are mean±SEM. (**D**) The Q101X mutation does not affect survival of female mice. Kaplan-Meier survival plot showing survival, *Tardbp^+/+^* n = 9 survival = 709±66 days, *Tardbp^+/Q101X^* n = 15, survival = 634±49 days (p = 0.235).

Grip-strength, rotarod performance and SHIRPA were assessed in male up to one year of age and in female mice up to two years of age. Longitudinal assessment of motor behaviour using grip-strength and rotarod tests revealed no abnormalities in *Tardbp^+/Q101X^* mice compared with wildtype controls, in both sexes up to 52 weeks of age and in females to 104 weeks (endpoint of the assessment) (**Figure 3A&3B**). There were no significant differences in bodyweight, measured every two weeks, over the lifespan of *Tardbp^+/+^* and *Tardbp^+/Q101X^* littermates (**Figure 3C**) and survival did not significantly differ between the two groups (*Tardbp^+/+^* survival = 709±66 days, n = 9; *Tardbp^+/Q101X^* survival = 634±49 days, n = 15; p = 0.235; **Figure 3D**). Known causes of premature death, such as cancer, did not differ between wildtype and *Tardbp^+/Q101X^* mice.

Additionally, as part of the behavioural testing, startle response and pre-pulse inhibition were assessed at 10 and 22 weeks, as well as open field at 14 and 30 weeks of age, but yielded no significant differences between wildtype and heterozygous mutant littermates (data not shown).

### Abnormal Limb-clasping and Body Tone in *Tardbp^+/Q101X^* Mice

**Figure 4:**
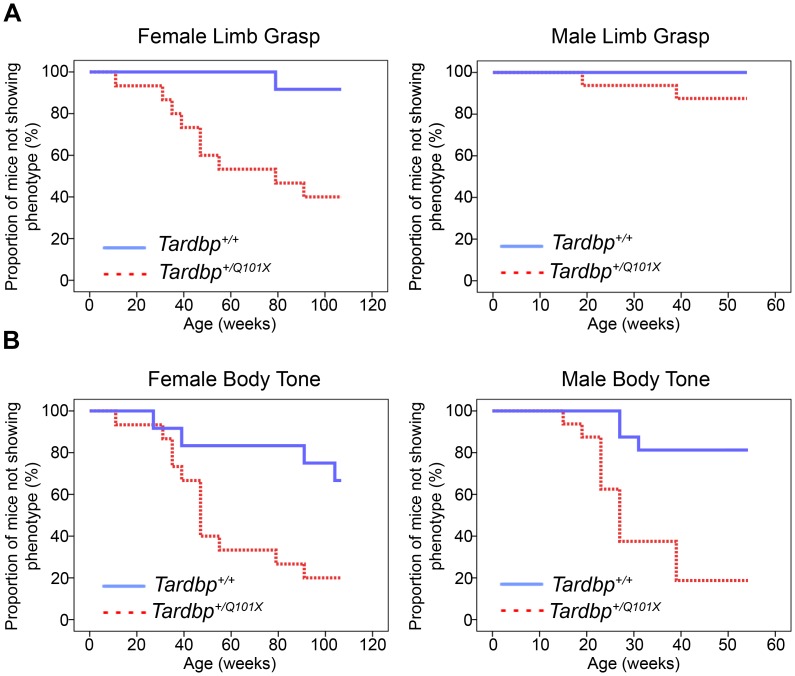
Limb-clasping and body tone abnormalities in *Tardbp^+/Q101X^* mice. (**A**) *Tardbp^+/Q101X^* mice develop hindlimb clasping. Plots show onset of hindlimb-clasping. The Y-axis is percentage of mice *not* showing the phenotype. At 7 weeks of age no mice showed limb clasping. Female mice (left panel): *Tardbp^+/+^* n = 12, *Tardbp^+/Q101X^* n = 15; Male mice (right panel): *Tardbp^+/+^* n = 16, *Tardbp^+/Q101X^* n = 16. Overall difference between *Tardbp^+/+^* and *Tardbp^+/Q101X^* p = 0.002 (Kaplan Meier log rank statistic), with a significant difference also noted between male and female *Tardbp^+/Q101X^* mice up to one year (p = 0.046). (**B**) Progressive development of abnormal body tone in male and female *Tardbp^+/Q101X^* mice. No mice displayed softer, abnormal body tone at 7 weeks of age. Male mice: *Tardbp^+/+^* n = 16, *Tardbp^+/Q101X^* n = 16. Female mice *Tardbp^+/+^* n = 12, *Tardbp^+/Q101X^* n = 15. *Tardbp^+/+^* versus *Tardbp^+/Q101X^* p<0.0001 (Kaplan Meier log rank statistic), with a trend in sex differences of *Tardbp^+/Q101X^* genotype up to one year of age, p = 0.07 (Kaplan Meier log rank statistic).

Although motor behaviour and survival were not affected in *Tardbp^+/Q101X^* mice, during the SHIRPA assessment significant differences from wildtype littermates were seen with two phenotypes: hindlimb-clasping and body tone (**Figure 4**). Hindlimb-clasping occured in 60% of *Tardbp^+/Q101X^* females by two years of age, n = 15, with an average onset of 61 weeks (**Figure 4A**). Male *Tardbp^+/Q101X^* mice did not show this phenotype to a significant extent; however they were not assessed beyond 52 weeks of age.

As well as limb-clasping, *Tardbp^+/Q101X^* mice also showed abnormal, softer, abdominal body tone, a component of the SHIRPA which is poorly reported in the literature. This is a highly penetrant phenotype, which manifested with age, and was seen in more than 80% of male and female *Tardbp^+/Q101X^* mice, with a mean age of onset in female *Tardbp^+/Q101X^* mice of 32 weeks of age (**Figure 4B**). In comparison, less than 50% of littermate control mice showed a soft body tone (3 of 16 male mice and 4 of 12 female mice at 52 weeks of age: overall 5 of 28). To determine whether the body tone phenotype was associated with reduced neuronal innervation, we examined neuromuscular junctions of abdominal muscles, including the external oblique muscle. No denervation or abnormal neuromuscular junctions were observed (**Figure S3**).

### No Evidence of Hindlimb Neuromuscular Dysfunction in *Tardbp^+/Q101X^* Mice at 18 Months of Age

Although no signs of motor dysfunction were evident in grip strength or rotarod performance of *Tardbp^+/Q101X^* mice, we conducted an *in vivo* assessment of hindlimb neuromuscular function, a more sensitive measure of functional decline than grip strength or rotarod tests, in a cohort of male mice at 18 months of age (*Tardbp^+/+^* n = 12, *Tardbp^+/Q101X^* n = 4).

Examples of isometric force recordings are shown in **Figure S4**. The mean maximum tetanic force of tibialis anterior (TA) and extensor digitorum longus (EDL) muscles are summarised in **Figure S4**, and muscle contraction, relaxation and fatigue characteristics, as well as EDL motor unit survival are summarised in **Figure S5**. No differences were seen between *Tardbp^+/+^* and *Tardbp^+/Q101X^* male mice in any parameter assessed (data in **Table S1)**.

Alongside neuromuscular assessment, brains from *Tardbp^+/+^* and *Tardbp^+/Q101X^* mice were examined for pathology at one year of age. No overt structural abnormalities were observed using a panel of markers against GFAP, IBA-1, p62 and MBP (data not shown).

### Does the *Tardbp^Q101X^* Mutation Affect Transgenic SOD1-ALS Mouse Phenotypes?

Since there is evidence to suggest that SOD1 and TDP43 may interact [20]–[22], and in order to establish whether motor dysfunction may manifest in *Tardbp^+/Q101X^* mice in the presence of an ALS-related stress, *Tardbp^+/Q101X^* mice were crossed with *SOD1^G93Adl^* transgenic mice which have been previously been shown to model ALS, including deterioration of hindlimb neuromuscular function and a shortened lifespan ranging between 31–38 weeks [64].

**Figure 5:**
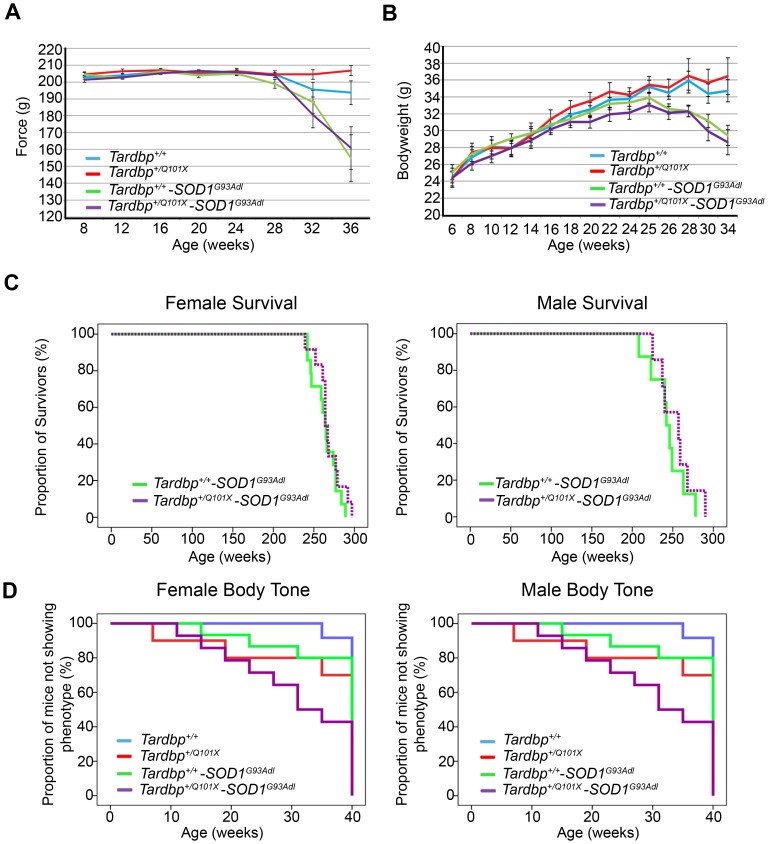
No interactive effects between *Tardbp^+/Q101X^* and *SOD1^G93Adl^* mice. (**A**) Grip strength of *SOD1^G93Adl^* mice (both sexes) is not affected by the Q101X mutation in *Tardbp*. *SOD1^G93Adl^* mice show progressive loss of grip strength (versus *Tardbp^+/+^* p = 0.002, LSD test from one-way ANOVA), however there is no difference between *Tardbp^+/+^*, *SOD1^G93Adl^* and *Tardbp^+/Q101X^*, *SOD1^G93Adl^* mice (p>0.05). Data are mean±SEM. (**B**) Progressive bodyweight loss in *SOD1^G93Adl^* mice is not affected by the Q101X mutation in *Tardbp*. Data are mean±SEM. (**C**) Survival plots for female (left panel) and male (right panel) mice show no effect of the Q101X mutation on survival of *SOD1^G93Adl^* mice. Endstage was defined as humane end-point or greater than 20% weight loss. *Tardbp^+/+^*, *SOD1^G93Adl^* 256±4 days n = 22, *Tardbp^+/Q101X^*, *SOD1^G93Adl^* 263±8 days n = 19 (total number including both sexes, with equal ratio), p = 0.227 (Kaplan Meier log rank statistic) (**D**) Body tone phenotype of *Tardbp^+/Q101X^* is replicated but not significantly different in *Tardbp^+/Q101X^*, *SOD1^G93Adl^* mice (female mice: left panel; male mice: right panel). Mean age at which body tone phenotype was shown for both sexes combined: *Tardbp^+/+^* = absent, *Tardbp^+/Q101X^* = 32 weeks of age (more than 50% of females), *Tardbp^+/+^*, *SOD1^G93Adl^* = absent, *Tardbp^+/Q101X^*, *SOD1^G93Adl^* n = 26 weeks (50% for both sexes). *Tardbp^+/+^*, *SOD1^G93Adl^* versus *Tardbp^+/Q101X^*, *SOD1^G93Adl^* p = 0.042, *Tardbp^+/Q101X^* versus *Tardbp^+/Q101X^*, *SOD1^G93Adl^* p = 0.982, *Tardbp^+/+^* versus *Tardbp^+/Q101X^* p = 0.006 (Kaplan Meier log rank statistic). Overall p = 0.008, with no significant difference between sexes within genotypes. Female mice: *Tardbp^+/+^* n = 10, *Tardbp^+/Q101X^* n = 5, *Tardbp^+/+^*, *SOD1^G93Adl^* n = 14, *Tardbp^+/Q101X^*, *SOD1^G93Adl^* n = 14. Male mice: *Tardbp^+/+^* n = 12, *Tardbp^+/Q101X^* n = 10, *Tardbp^+/+^*, *SOD1^G93Adl^* n = 15, *Tardbp^+/Q101X^*, *SOD1^G93Adl^* n = 14.

Progeny from this cross were assessed from 7 weeks of age by SHIRPA, grip-strength and rotarod tests, all of which become progressively more abnormal in *SOD1^G93Adl^* mice [63]. Although phenotypes previously seen in *Tardbp^+/Q101X^* and *SOD1^G93Adl^* mice were replicated in the equivalent progeny, no genetic interaction effects were observed in *Tardbp^+/Q101X^*, *SOD1^G93Adl^* double mutant offspring (**Figure 5**), i.e. the mutant offspring were not significantly more badly affected than their *SOD1^G93Adl^* littermates, other than for two relatively minor effects on (1) TA muscle relaxation time, and (2) EDL muscle weight, as below. Deficits in grip-strength, bodyweight and survivalinduced by the *SOD1* transgene were not affected by the Q101X mutation (**Figure 5A, B and C**). The body tone phenotype observed in *Tardbp^+/Q101X^* mice was replicated in *Tardbp^+/Q101X^*, *SOD1^G93Adl^* mice, which had a mean age of onset of 26 weeks, and this was not exacerbated by the *SOD1^G93A^* transgene (**Figure 5D**).

Physiological assessment of isometric muscle force in TA and EDL hindlimb muscles of 32-33-week old male mice (*Tardbp^+/+^* n = 9, *Tardbp^+/Q101X^* n = 6, *Tardbp^+/+^*, *SOD1^G93Adl^* n = 7, *Tardbp^+/Q101X^*, *SOD1^G93Adl^* n = 7), a symptomatic stage of disease in *SOD1^G93Adl^* animals, showed that expression of *Tardbp^+/Q101X^* had no effect on mean tetanic muscle force (**Figure S6A, B**). Assessment of TA contraction and relaxation characteristics did not reveal any changes in the TTP of TA muscles (**Figure S6C**). However, relaxation of TA muscles was significantly slower in *Tardbp^+/Q101X^*, *SOD1^G93Adl^* mice compared to *Tardbp^+/+^*, *SOD1^G93Adl^* mice (42.0 ms±3.5 ms n = 10, and 30.4 ms±2.8 ms n = 10, respectively, p = 0.007; **Figure S6D**) but did not differ between *Tardbp^+/+^* and *Tardbp^+/Q101X^* mice. Contraction times of EDL muscles did not differ between groups (**Figure S6E**). Although relaxation time of the EDL was not significantly different between *Tardbp^+/+^*, *SOD1^G93Adl^* and *Tardbp^+/Q101X^*, *SOD1^G93Adl^* littermates, a significant difference was evident between *Tardbp^+/+^* and *Tardbp^+/Q101X^* mice (21.4 ms±1.8 ms n = 7, and, 15.9 ms±0.7 ms n = 9, p<0.05), albeit not in a manner which would suggest dysfunction - slower relaxation times are associated with neuromuscular dysfunction (**Figure S6F**). We note we did not see this difference in previous studies comparing just *Tardbp^+/+^* and *Tardbp^+/Q101X^* littermates. No differences were observed in the fatigue characteristics between groups (**Figure S6G**). The number of surviving EDL motor units was also assessed and did not reveal significant differences between any groups (**Figure S6H;** all data also presented in **Table S2**).

TA and EDL weights were also recorded. TA weight did not differ between *Tardbp^+/+^* and *Tardbp^+/Q101X^* mice, but was reduced in *Tardbp^+/Q101X^*, *SOD1^G93Adl^* mice compared to *Tardbp^+/+^*, *SOD1^G93Adl^* littermates, although this difference was not statistically significant (41.7g±1.2 mg, n = 12 and 46.3 mg±1.9 mg, n = 9, respectively; p = 0.051; **Figure S7A**). EDL muscle weight did not differ between *Tardbp^+/+^* and *Tardbp^+/Q101X^* mice, however a significant reduction was seen in *Tardbp^+/Q101X^*, *SOD1^G93Adl^* mice compared to *Tardbp^+/+^*, *SOD1^G93Adl^* mice (10.1 mg±0.7 mg, n = 8 and 12.6 mg±0.9 mg, n = 8, respectively; p = 0.035; **Figure S7B**).

Finally, no differences were seen in the number of motor neurons in the sciatic motor pool of *Tardbp^+/+^*, *SOD1^G93Adl^* and *Tardbp^+/Q101X^*, *SOD1^G93Adl^* mice (**Figure S7C**).

Taken together, these findings show that the Q101X TDP43 mutation does not significantly affect the hindlimb neuromuscular phenotype of *SOD1^G93Adl^* mice.

## Discussion

We have carried out a comprehensive assessment of a novel mutant *Tardbp* mouse with a nonsense mutation predicted to cause a truncation in the N-terminus of TDP43. Characterisation of the *Tardbp^Q101X^* strain has revealed novel molecular and behavioural phenotypic changes that will help to shed more light on the normal role and pathophysioglogy of TDP43.

We found Q101X mutation was homozygous embryonic lethal and *Tardbp^Q101X/Q101X^* embryos died *in utero* before E6.5. This result indicates a likely loss of function and adds further support for a crucial role for TDP43 during embryonic development; complete deficiency of TDP43 is embryonic lethal [27]–[29].

Heterozygous *Tardbp^+/Q101X^* mice had a decrease in the relative abundance of mutant compared to wildtype transcripts. This may arise from either non-sense mediated decay of mutant mRNA due to the presence of an early stop codon or downregulation of expression from the mutant allele. Further investigation may shed more light on *Tardbp* autoregulation mechanisms [25], [48], [49] as wildtype transcripts (likely to be full-length) were at similar levels between wildtype and heterozygous mutant littermates.

Full-length TDP43 protein levels are equivalent between *Tardbp^+/+^* and *Tardbp^+/Q101X^* littermates, and we could not detect truncated Q101X protein in brain from aged heterozygous mice. Thus, although we did not look during development, or in all tissues, we might expect these heterozygotes to be normal. Curiously, however, assessment of TDP43 alternative splicing function in *Tardbp^+/Q101X^* mice showed aberrant exon inclusion in two of five gene targets tested, and *Tardbp^+/Q101X^* mice developed hindlimb-clasping and an intriguing body tone phenotype. A number of possibilities could explain these phenotypes, including: (1) The upregulation of the wildtype transcripts in response to the null allele (Q101X) may not reach totally normal levels as in *Tardbp^+/+^* mice and small, undetectable differences, perhaps in particular cellular populations, may give rise to these phenotypes and/or (2) the mutant transcript or very low levels of truncated protein may interfere with normal TDP43 function.

Exon inclusion of two targets, *Sort1* and *Pdp1*, was altered in the same manner as that following TDP43 depletion [25], therefore suggesting a partial loss-of-function effect of the TDP43 Q101X mutation. However, cassette exon inclusion/exclusion of 3 other target genes: *Dnajc5*, *Kcnd5* and *Kcnip2*, did not differ between *Tardbp^+/+^* and *Tardbp^+/Q101X^* mice. Moreover, neurite outgrowth, a process also known to be affected by loss of TDP43 function, was not affected in primary embryonic motor neurons from *Tardbp^+/Q101X^* mice. It is unclear why cassette exon inclusion/exclusion of *Sort1* and *Pdp1* are affected yet other targets are not, although this could perhaps indicate differential dependence on TDP43 for regulation of alternative splicing.

Hindlimb clasping is often observed in rodent models of neuromuscular disease including ALS and has been associated with lower motor neuron degeneration, although it can also be caused by muscular dystrophy [72] and loss of cortical neurons [73]. It has also been noted following motor neuron-specific depletion of TDP43 [52]. Pathological analysis of brains from *Tardbp^+/Q101X^* mice did not reveal overt signs of degeneration and *in vivo* assessment of hindlimb neuromuscular function did not suggest lower motor neuron degeneration at 18 months of age.

When body tone of wildtype mice is examined, a basal muscular tone with a reflexive elicited response is normally felt by the assessor [74]. Loss of body tone may therefore be due to muscular alterations or dysfunction of the neuronal reflexive control. Body tone differences have been reported in rat [75] and mouse studies [76], [77]. Reduced body tone was detected in Diazepam treated mice [77], which also affected mouse posture by reducing muscle tone in the abdomen and limbs. Mice treated with the acetylcholine esterase inhibitor donepezil also showed a body tone reduction alongside multiple toxic effects ranging from motor deficits through to decreased arousal and piloerection [76]. In these reports, body tone reduction is part of a wider array of phenotypes which are the result of muscular and neurological effects. Clearly this phenotype warrants further investigation to identify the cellular pathology in *Tardbp^+/Q101X^* mice.

In the *Tardbp* ^+/Q101X^ mice, the softer body tone and limb- clasping are the only phenotypes detected, and mice may have subtle slowly-progressing neuromuscular deficits that remain mild throughout life. Thus, possible loss-of-function effects in *Tardbp^+/Q101X^* mice may manifest as subtle neurological dysfunction rather neuronal loss. Consistent with this possibility, neuronal atrophy, but crucially not loss, has been observed in adult mice with conditional knockout of *Tardbp* in motor neurons [53]. Furthermore, heterozygous knockout (*Tardbp* ^+/−^) mice displayed motor deficits on an inverted grid test but without evidence of pathological changes in motor neurons and, as here, TDP43 protein levels auto-regulated to match those of wildtype littermates [27]. Thus potentially, *Tardbp* ^+/−^ and *Tardbp* ^+/Q101X^ mice may share similar perturbations resulting in muscular and neurological alterations, although no motor deficits were detected in *Tardbp* ^+/Q101X^ mice showing that they likely model differing aspects of TDP43 biology.

As TDP43 and SOD1 have been proposed to interact [20]–[22], we investigated progeny of crossing *Tardbp^+/Q101X^* mice with the *SOD1^G93Adl^* transgenic model of ALS. This *SOD1*-ALS model was chosen in preference to the more commonly used SOD1^G93A^ mouse, in which ALS-like symptoms are seen much earlier and progress more rapidly [68], so we could identify subtle effects. The TDP43 mutation did not affect survival, bodyweight loss or disease course in *Tardbp^+/Q101X^*, *SOD1^G93Adl^* progeny compared to *SOD1^G93Adl^* littermates. However, TA relaxation time was slower in the double mutants and more extensive atrophy of the EDL muscle was found, albeit in the absence of significant changes in muscle force or motor unit survival, compared to *SOD1^G93Adl^* littermates. Taken together, these results show that although the Q101X mutation partially disrupts TDP43 function, it does not notably modify the ALS-phenotype of *SOD1^G93Adl^* mice. These findings support published work which was unable to detect any interaction between SOD1 and TDP43 in a *C.elegans* model of ALS [24].

In summary, the first ENU-induced point mutant *Tardbp* mouse, the *Tardbp^Q101X^* strain, provides further evidence for the important role of TDP43 in development and for autoregulation of TDP43. *Tardbp^+/Q101X^* mice have a likely partial loss-of-function phenotype of exon inclusion of selected targets, and these mice are a useful new genetic model for investigating TDP43 function *in vivo*. Moreover, *Tardbp^+/Q101X^* mice develop abnormal hindlimb and body tone phenotypes, in the absence of overt neurodegeneration. The underlying cause of these phenotypes is remains unknown and may indicate previously unknown functions of TDP43. We note that this new mouse strain is freely available for the community to further investigate TDP43 biology.

## Supporting Information

Figure S1 **TDP43 protein levels do not change in** ***Tardbp^+/Q101X^*** **mice.** **(A, B)** N-terminal anti TDP-43 antibodies do not show any novel truncated TDP-43 band in *Tardbp^+/Q101X^* soluble or RIPA-insoluble brain fractions from 18 month-old males. N-terminal antibodies used were: (**A**) Cosmo Bio (CAC-TIP-TD-P07) and (**B**) Abcam (ab50930). (**C**) No significant differences in full-length TDP43 protein levels relative to tubulin between *Tardbp^+/+^* (0.99±0.20) and *Tardbp^+/Q101X^* (0.76±0.14) using an antibody directed against C-terminus of TDP43 (Proteintech 12892-1-AP). TDP43 levels (green) were assessed from RIPA soluble (p = 0.375) and RIPA-insoluble fractions (p = 0.123) from whole spinal cord lysates using 3 mice per genotype at 18 months of age. Tubulin (red) was used as a loading control. Data are mean±SEM.
(TIF)

Figure S2 **Neurite outgrowth of primary embryonic motor neurons is not affected by the** ***Tardbp*** **^Q101X^ mutation.** **(A)** Representative images of primary embryonic motor neurons stained for the neuronal marker B-III tubulin (red) and DAPI (blue) from *Tardbp^+/+^* and *Tardbp^+/Q101X^* embryos. **(B)** Mean longest neurite length was not significantly different between *Tardbp^+/+^* and *Tardbp^+/Q101X^* motor neurons (n = 175 and n = 117 neurons, respectively). **(C)** Neither were any differences seen between mean neurite length of *Tardbp^+/+^* and *Tardbp^+/Q101X^* motor neurons. Data are mean±SEM from 3 independent experiments.
(TIF)

Figure S3 ***Tardbp*** **^Q101X^ mice present a normal innervation pattern of the external abdominal oblique muscle.** **(A, B)** Representative immunofluorescence images of whole mount abdominal oblique muscle from *Tardbp^+/+^* **(A)** and *Tardbp^+/Q101X^* **(B)** mice at ∼1 year of age. A normal innervation pattern was present for both genotypes. Postsynaptic, presynaptic and axonal regions were identified by acetylcholine receptor (red), synaptic vesicle protein (green) and neurofilament (green) staining, respectively. Scale bar represent 50 µm in both images.
(TIF)

Figure S4 **Examples of recordings from** ***in vivo*** **assessment of neuromuscular function of male** ***Tardbp^+/+^*** **mice at 18 months of age.** **(A)** Example recording of maximum twitch (smaller peak) and tetanic force (larger peak) from TA muscle. **(B)** Example trace from an EDL muscle illustrating how contraction time (TTP) and relaxation time (½RT) are calculated from maximum twitch force recordings. **(C)** Example trace demonstrating motor unit number estimation of the EDL. **(D)** Trace recording of fatigue characteristic of an EDL muscle, where the fatigue index (FI) is calculated as the ratio of force after 180 seconds (F_180_) compared to initial force (F_0_).
(TIF)

Figure S5 **No evidence of neuromuscular dysfunction in male** ***Tardbp*** **^+/Q101X^ mice at 18 months of age.** **(A)** Maximum tetanic force recorded from TA muscles was not significantly different between *Tardbp^+/+^* (n = 12 muscles) and *Tardbp^+/Q101X^* (n = 6) mice. **(B)** No difference was seen in the maximum tetanic force of EDL muscles in *Tardbp^+/+^* (n = 8) and *Tardbp^+/Q101X^* (n = 5) mice. **(C&D)** Assessment of TA contraction (TTP) and relaxation (½RT) characteristics in *Tardbp^+/+^* and *Tardbp^+/Q101X^* mice (n = 11, and n = 6, respectively) did not reveal any significant differences. **(E&F)** EDL muscle contraction (TTP) and relaxation (½RT) characteristics did not differ between *Tardbp^+/+^* and *Tardbp^+/Q101X^* mice (n = 8 and n = 5, respectively) **(G)** A fatigue index of EDL muscles was established but did not show any difference between *Tardbp^+/+^* (n = 7) and *Tardbp^+/Q101X^* (n = 3) mice. **(H)** The number of motor units innervating EDL muscles was assessed but also failed to show any difference between *Tardbp^+/+^* (n = 8) and *Tardbp^+/Q101X^* (n = 4) mice.
(TIF)

Figure S6 **Assessment of hindlimb neuromuscular function in male** ***Tardbp^+/Q101X^*** **,** ***SOD1^G93Adl^*** **mice.** **(A)** Maximum tetanic force recorded from the TA was not significantly different between *Tardbp^+/+^* and *Tardbp^+/Q101X^* mice (both n = 10), or between *Tardbp^+/+^*, *SOD1^G93Adl^* and *Tardbp^+/Q101X^*, *SOD1^G93Adl^* mice (both n = 10). **(B)** EDL tetanic force did not differ between *Tardbp^+/+^* (n = 8) and *Tardbp^+/Q101X^* (n = 9) mice, or between *Tardbp^+/+^*, *SOD1^G93Adl^* (n = 10) and *Tardbp^+/Q101X^*, *SOD1^G93Adl^* (n = 9) mice. **(C&D)** Contraction (TTP) and relaxation (½RT) of TA muscles did not differ between *Tardbp^+/+^* (n = 9 and n = 8) and *Tardbp^+/Q101X^* (n = 13, n = 12) mice, and although TTP did not differ between *Tardbp^+/+^*, *SOD1^G93Adl^* (n = 9) and *Tardbp^+/Q101X^*, *SOD1^G93Adl^* (n = 10) mice, relaxation time was significantly slower in *Tardbp^+/Q101X^*, *SOD1^G93Adl^* mice compared to *Tardbp^+/+^*, *SOD1^G93Adl^* mice (both n = 10; p = 0.007). **(E)** Contraction of EDL muscles was not different between *Tardbp^+/+^* (n = 8) and *Tardbp^+/Q101X^* mice (n = 9) or between *Tardbp^+/+^*, *SOD1^G93Adl^* (n = 10) and *Tardbp^+/Q101X^*, *SOD1^G93Adl^* (n = 10) mice. **(F)** EDL relaxation time was significantly quicker in *Tardbp^+/Q101X^* (n = 9) mice compared to *Tardbp^+/+^* mice (n = 7; p<0.05), however no difference was observed between *Tardbp^+/+^*, *SOD1^G93Adl^* (n = 10) and *Tardbp^+/Q101X^*, *SOD1^G93Adl^* (n = 10) mice. **(G)** Fatigue characteristics of the EDL, defined as the FI, did not differ between *Tardbp^+/+^* (n = 9) and *Tardbp^+/Q101X^* (n = 8) mice, or between *Tardbp^+/+^*, *SOD1^G93Adl^* (n = 7) and *Tardbp^+/Q101X^*, *SOD1^G93Adl^* (n = 7) mice. **(H)** The number of surviving motor units of EDL muscles was not significantly different between *Tardbp^+/+^* (n = 8) and *Tardbp^+/Q101X^* (n = 8) mice, or between *Tardbp^+/+^*, *SOD1^G93Adl^* (n = 8) and *Tardbp^+/Q101X^*, *SOD1^G93Adl^* (n = 10) mice.
(TIF)

Figure S7 **Muscle weights and motor neuron survival in male** ***Tardbp^+/+^*** **,** ***SOD1^G93Adl^*** **and** ***Tardbp^+/Q101X^*** **,** ***SOD1^G93Adl^*** **mice at 32–33 weeks of age.** **(A)** No difference between TA muscle weights of *Tardbp^+/+^* (n = 12) and *Tardbp^+/Q101X^* (n = 17) mice, or between *Tardbp^+/+^*, *SOD1^G93Adl^* (n = 9) and *Tardbp^+/Q101X^*, *SOD1^G93Adl^* (n = 12) mice (p = 0.051). **(B)** EDL muscle weight was similar between *Tardbp^+/+^* (n = 12) and *Tardbp^+/Q101X^* (n = 17) mice, but was showed a significant difference between *Tardbp^+/+^*, *SOD1^G93Adl^* (n = 8) and *Tardbp^+/Q101X^*, *SOD1^G93Adl^* (n = 8) mice (p = 0.035). **(C)** The number of motor neurons of the sciatic pool (L2-L6) was counted in *Tardbp^+/+^*, *SOD1^G93Adl^* (n = 3) and *Tardbp^+/Q101X^*, *SOD1^G93Adl^* (n = 4) mice and did not reveal any differences.
(TIF)

Table S1 Normal hindlimb neuromuscular function in *Tardbp^+/Q101X^* male mice at 18 months of age.
(DOCX)

Table S2 The Q101X mutation in TDP43 does not affect neuromuscular function in 32–33 week old *SOD1^G93Adl^* mice.
(DOCX)

## References

[1] Robberecht W, Philips T (2013) The changing scene of amyotrophic lateral sclerosis. Nat Rev Neurosci 14: 248–264 10.1038/nrn3430 23463272

[2] Chen S, Sayana P, Zhang X, Le W (2013) Genetics of amyotrophic lateral sclerosis: an update. Mol Neurodegener 8: 28 10.1186/1750-1326-8-28 23941283 PMC3766231

[3] Al-Chalabi A, Jones A, Troakes C, King A, Al-Sarraj S, et al (2012) The genetics and neuropathology of amyotrophic lateral sclerosis. Acta Neuropathol (Berl) 124: 339–352 10.1007/s00401-012-1022-4 22903397

[4] Arai T, Hasegawa M, Akiyama H, Ikeda K, Nonaka T, et al (2006) TDP-43 is a component of ubiquitin-positive tau-negative inclusions in frontotemporal lobar degeneration and amyotrophic lateral sclerosis. Biochem Biophys Res Commun 351: 602–611 10.1016/j.bbrc.2006.10.093 17084815

[5] Mackenzie IRA, Bigio EH, Ince PG, Geser F, Neumann M, et al (2007) Pathological TDP-43 distinguishes sporadic amyotrophic lateral sclerosis from amyotrophic lateral sclerosis with SOD1 mutations. Ann Neurol 61: 427–434 10.1002/ana.21147 17469116

[6] Neumann M, Sampathu DM, Kwong LK, Truax AC, Micsenyi MC, et al (2006) Ubiquitinated TDP-43 in frontotemporal lobar degeneration and amyotrophic lateral sclerosis. Science 314: 130–133 10.1126/science.1134108 17023659

[7] Borroni B, Bonvicini C, Alberici A, Buratti E, Agosti C, et al (2009) Mutation within TARDBP leads to frontotemporal dementia without motor neuron disease. Hum Mutat 30: E974–983 10.1002/humu.21100 19655382

[8] Kabashi E, Valdmanis PN, Dion P, Spiegelman D, McConkey BJ, et al (2008) TARDBP mutations in individuals with sporadic and familial amyotrophic lateral sclerosis. Nat Genet 40: 572–574 10.1038/ng.132 18372902

[9] Sreedharan J, Blair IP, Tripathi VB, Hu X, Vance C, et al (2008) TDP-43 mutations in familial and sporadic amyotrophic lateral sclerosis. Science 319: 1668–1672 10.1126/science.1154584 18309045 PMC7116650

[10] Chiò A, Calvo A, Moglia C, Restagno G, Ossola I, et al (2010) Amyotrophic lateral sclerosis-frontotemporal lobar dementia in 3 families with p.Ala382Thr TARDBP mutations. Arch Neurol 67: 1002–1009 10.1001/archneurol.2010.173 20697052 PMC3535689

[11] Borroni B, Archetti S, Del Bo R, Papetti A, Buratti E, et al (2010) TARDBP mutations in frontotemporal lobar degeneration: frequency, clinical features, and disease course. Rejuvenation Res 13: 509–517 10.1089/rej.2010.1017 20645878

[12] DeJesus-Hernandez M, Mackenzie IR, Boeve BF, Boxer AL, Baker M, et al (2011) Expanded GGGGCC hexanucleotide repeat in noncoding region of C9ORF72 causes chromosome 9p-linked FTD and ALS. Neuron 72: 245–256 10.1016/j.neuron.2011.09.011 21944778 PMC3202986

[13] Renton AE, Majounie E, Waite A, Simón-Sánchez J, Rollinson S, et al (2011) A hexanucleotide repeat expansion in C9ORF72 is the cause of chromosome 9p21-linked ALS-FTD. Neuron 72: 257–268 10.1016/j.neuron.2011.09.010 21944779 PMC3200438

[14] Millecamps S, Salachas F, Cazeneuve C, Gordon P, Bricka B, et al (2010) SOD1, ANG, VAPB, TARDBP, and FUS mutations in familial amyotrophic lateral sclerosis: genotype-phenotype correlations. J Med Genet 47: 554–560 10.1136/jmg.2010.077180 20577002

[15] Chiò A, Calvo A, Mazzini L, Cantello R, Mora G, et al (2012) Extensive genetics of ALS A population-based study in Italy. Neurology 79: 1983–1989 10.1212/WNL.0b013e3182735d36 23100398 PMC3484987

[16] Majounie E, Renton AE, Mok K, Dopper EGP, Waite A, et al (2012) Frequency of the C9orf72 hexanucleotide repeat expansion in patients with amyotrophic lateral sclerosis and frontotemporal dementia: a cross-sectional study. Lancet Neurol 11: 323–330 10.1016/S1474-4422(12)70043-1 22406228 PMC3322422

[17] Cruts M, Gijselinck I, Van Langenhove T, van der Zee J, Van Broeckhoven C (n.d.) Current insights into the C9orf72 repeat expansion diseases of the FTLD/ALS spectrum. Trends Neurosci. Available: http://www.sciencedirect.com/science/article/pii/S0166223613000842. Accessed 25 June 2013. 10.1016/j.tins.2013.04.010 23746459

[18] Arai T, Mackenzie IRA, Hasegawa M, Nonoka T, Niizato K, et al (2009) Phosphorylated TDP-43 in Alzheimer’s disease and dementia with Lewy bodies. Acta Neuropathol (Berl) 117: 125–136 10.1007/s00401-008-0480-1 19139911

[19] Hasegawa M, Arai T, Akiyama H, Nonaka T, Mori H, et al (2007) TDP-43 is deposited in the Guam parkinsonism-dementia complex brains. Brain J Neurol 130: 1386–1394 10.1093/brain/awm065 17439983

[20] Somalinga BR, Day CE, Wei S, Roth MG, Thomas PJ (2012) TDP-43 identified from a genome wide RNAi screen for SOD1 regulators. PloS One 7: e35818 10.1371/journal.pone.0035818 22563406 PMC3338536

[21] Pokrishevsky E, Grad LI, Yousefi M, Wang J, Mackenzie IR, et al (2012) Aberrant Localization of FUS and TDP43 Is Associated with Misfolding of SOD1 in Amyotrophic Lateral Sclerosis. PLoS ONE 7: e35050 10.1371/journal.pone.0035050 22493728 PMC3320864

[22] Volkening K, Leystra-Lantz C, Yang W, Jaffee H, Strong MJ (2009) Tar DNA binding protein of 43 kDa (TDP-43), 14-3-3 proteins and copper/zinc superoxide dismutase (SOD1) interact to modulate NFL mRNA stability. Implications for altered RNA processing in amyotrophic lateral sclerosis (ALS). Brain Res 1305: 168–182 10.1016/j.brainres.2009.09.105 19815002

[23] Belzil VV, Daoud H, Dion PA, Rouleau GA (2011) No effect on SOD1 splicing by TARDP or FUS mutations. Arch Neurol 68: 395–396 10.1001/archneurol.2011.1 21403029

[24] Kabashi E, Bercier V, Lissouba A, Liao M, Brustein E, et al (2011) FUS and TARDBP but not SOD1 interact in genetic models of amyotrophic lateral sclerosis. PLoS Genet 7: e1002214 10.1371/journal.pgen.1002214 21829392 PMC3150442

[25] Polymenidou M, Lagier-Tourenne C, Hutt KR, Huelga SC, Moran J, et al (2011) Long pre-mRNA depletion and RNA missplicing contribute to neuronal vulnerability from loss of TDP-43. Nat Neurosci 14: 459–468 10.1038/nn.2779 21358643 PMC3094729

[26] Budini M, Baralle FE, Buratti E (2011) Regulation of gene expression by TDP-43 and FUS/TLS in frontotemporal lobar degeneration. Curr Alzheimer Res 8: 237–245. 21222602 10.2174/156720511795563719

[27] Kraemer BC, Schuck T, Wheeler JM, Robinson LC, Trojanowski JQ, et al (2010) Loss of murine TDP-43 disrupts motor function and plays an essential role in embryogenesis. Acta Neuropathol (Berl) 119: 409–419 10.1007/s00401-010-0659-0 20198480 PMC2880609

[28] Sephton CF, Good SK, Atkin S, Dewey CM, Mayer P 3rd, et al (2010) TDP-43 is a developmentally regulated protein essential for early embryonic development. J Biol Chem 285: 6826–6834 10.1074/jbc.M109.061846 20040602 PMC2825476

[29] Wu L-S, Cheng W-C, Hou S-C, Yan Y-T, Jiang S-T, et al (2010) TDP-43, a neuro-pathosignature factor, is essential for early mouse embryogenesis. Genes New York N 2000 48: 56–62 10.1002/dvg.20584 20014337

[30] Sephton CF, Cenik B, Cenik BK, Herz J, Yu G (2012) TDP-43 in central nervous system development and function: clues to TDP-43-associated neurodegeneration. Biol Chem 393: 589–594 10.1515/hsz-2012-0115 22944662 PMC3537500

[31] Moisse K, Volkening K, Leystra-Lantz C, Welch I, Hill T, et al (2009) Divergent patterns of cytosolic TDP-43 and neuronal progranulin expression following axotomy: implications for TDP-43 in the physiological response to neuronal injury. Brain Res 1249: 202–211 10.1016/j.brainres.2008.10.021 19046946

[32] Parker SJ, Meyerowitz J, James JL, Liddell JR, Crouch PJ, et al (2012) Endogenous TDP-43 localized to stress granules can subsequently form protein aggregates. Neurochem Int 60: 415–424 10.1016/j.neuint.2012.01.019 22306778

[33] Higashi S, Kabuta T, Nagai Y, Tsuchiya Y, Akiyama H, et al. (2013) TDP-43 associates with stalled ribosomes and contributes to cell survival during cellular stress. J Neurochem. doi:10.1111/jnc.12194. 23398327

[34] Swarup V, Audet J-N, Phaneuf D, Kriz J, Julien J-P (2012) Abnormal regenerative responses and impaired axonal outgrowth after nerve crush in TDP-43 transgenic mouse models of amyotrophic lateral sclerosis. J Neurosci Off J Soc Neurosci 32: 18186–18195 10.1523/JNEUROSCI.2267-12.2012 PMC6621743 23238732

[35] Li YR, King OD, Shorter J, Gitler AD (2013) Stress granules as crucibles of ALS pathogenesis. J Cell Biol 201: 361–372 10.1083/jcb.201302044 23629963 PMC3639398

[36] Dewey CM, Cenik B, Sephton CF, Johnson BA, Herz J, et al (2012) TDP-43 aggregation in neurodegeneration: are stress granules the key? Brain Res 1462: 16–25 10.1016/j.brainres.2012.02.032 22405725 PMC3372581

[37] Fiesel FC, Schurr C, Weber SS, Kahle PJ (2011) TDP-43 knockdown impairs neurite outgrowth dependent on its target histone deacetylase 6. Mol Neurodegener 6: 64 10.1186/1750-1326-6-64 21878116 PMC3170629

[38] Fallini C, Bassell GJ, Rossoll W (2012) The ALS disease protein TDP-43 is actively transported in motor neuron axons and regulates axon outgrowth. Hum Mol Genet 21: 3703–3718 10.1093/hmg/dds205 22641816 PMC3406762

[39] Buratti E, Baralle FE (2012) TDP-43: gumming up neurons through protein-protein and protein-RNA interactions. Trends Biochem Sci 37: 237–247 10.1016/j.tibs.2012.03.003 22534659

[40] Cohen TJ, Lee VMY, Trojanowski JQ (2011) TDP-43 functions and pathogenic mechanisms implicated in TDP-43 proteinopathies. Trends Mol Med 17: 659–667 10.1016/j.molmed.2011.06.004 21783422 PMC3202652

[41] Fiesel FC, Kahle PJ (2011) TDP-43 and FUS/TLS: cellular functions and implications for neurodegeneration. FEBS J 278: 3550–3568 10.1111/j.1742-4658.2011.08258.x 21777389

[42] Lee EB, Lee VM-Y, Trojanowski JQ (2012) Gains or losses: molecular mechanisms of TDP43-mediated neurodegeneration. Nat Rev Neurosci 13: 38–50 10.1038/nrn3121 PMC3285250 22127299

[43] Xu Z-S (2012) Does a loss of TDP-43 function cause neurodegeneration? Mol Neurodegener 7: 27 10.1186/1750-1326-7-27 22697423 PMC3419078

[44] McGoldrick P, Joyce PI, Fisher EMC, Greensmith L (2013) Rodent models of amyotrophic lateral sclerosis. Biochim Biophys Acta. doi:10.1016/j.bbadis.2013.03.012. 23524377

[45] Joyce PI, Fratta P, Fisher EMC, Acevedo-Arozena A (2011) SOD1 and TDP-43 animal models of amyotrophic lateral sclerosis: recent advances in understanding disease toward the development of clinical treatments. Mamm Genome Off J Int Mamm Genome Soc 22: 420–448 10.1007/s00335-011-9339-1 21706386

[46] Tsao W, Jeong YH, Lin S, Ling J, Price DL, et al (2012) Rodent models of TDP-43: recent advances. Brain Res 1462: 26–39 10.1016/j.brainres.2012.04.031 22608070 PMC3613131

[47] Chiang P-M, Ling J, Jeong YH, Price DL, Aja SM, et al (2010) Deletion of TDP-43 down-regulates Tbc1d1, a gene linked to obesity, and alters body fat metabolism. Proc Natl Acad Sci U S A 107: 16320–16324 10.1073/pnas.1002176107 20660762 PMC2941284

[48] Ayala YM, De Conti L, Avendaño-Vázquez SE, Dhir A, Romano M, et al (2011) TDP-43 regulates its mRNA levels through a negative feedback loop. EMBO J 30: 277–288 10.1038/emboj.2010.310 21131904 PMC3025456

[49] Avendaño-Vázquez SE, Dhir A, Bembich S, Buratti E, Proudfoot N, et al (2012) Autoregulation of TDP-43 mRNA levels involves interplay between transcription, splicing, and alternative polyA site selection. Genes Dev 26: 1679–1684 10.1101/gad.194829.112 22855830 PMC3418585

[50] Budini M, Buratti E (2011) TDP-43 autoregulation: implications for disease. J Mol Neurosci MN 45: 473–479 10.1007/s12031-011-9573-8 21681666

[51] Buratti E, Baralle FE (2011) TDP-43: new aspects of autoregulation mechanisms in RNA binding proteins and their connection with human disease. FEBS J 278: 3530–3538 10.1111/j.1742-4658.2011.08257.x 21777388

[52] Wu L-S, Cheng W-C, Shen C-KJ (2012) Targeted depletion of TDP-43 expression in the spinal cord motor neurons leads to the development of amyotrophic lateral sclerosis-like phenotypes in mice. J Biol Chem 287: 27335–27344 10.1074/jbc.M112.359000 22718760 PMC3431639

[53] Iguchi Y, Katsuno M, Niwa J-I, Takagi S, Ishigaki S, et al (2013) Loss of TDP-43 causes age-dependent progressive motor neuron degeneration. Brain J Neurol 136: 1371–1382 10.1093/brain/awt029 23449777

[54] Arnold ES, Ling S-C, Huelga SC, Lagier-Tourenne C, Polymenidou M, et al (2013) ALS-linked TDP-43 mutations produce aberrant RNA splicing and adult-onset motor neuron disease without aggregation or loss of nuclear TDP-43. Proc Natl Acad Sci U S A 110: E736–745 10.1073/pnas.1222809110 23382207 PMC3581922

[55] Igaz LM, Kwong LK, Lee EB, Chen-Plotkin A, Swanson E, et al (2011) Dysregulation of the ALS-associated gene TDP-43 leads to neuronal death and degeneration in mice. J Clin Invest 121: 726–738 10.1172/JCI44867 21206091 PMC3026736

[56] Acevedo-Arozena A, Wells S, Potter P, Kelly M, Cox RD, et al (2008) ENU mutagenesis, a way forward to understand gene function. Annu Rev Genomics Hum Genet 9: 49–69. 18949851 10.1146/annurev.genom.9.081307.164224

[57] Hrabé de Angelis MH, Flaswinkel H, Fuchs H, Rathkolb B, Soewarto D, et al (2000) Genome-wide, large-scale production of mutant mice by ENU mutagenesis. Nat Genet 25: 444–447 10.1038/78146 10932192

[58] Rathkolb B, Fuchs E, Kolb HJ, Renner-Müller I, Krebs O, et al (2000) Large-scale N-ethyl-N-nitrosourea mutagenesis of mice-from phenotypes to genes. Exp Physiol 85: 635–644. 11187959

[59] Aizawa-Abe M, Ebihara K, Ebihara C, Mashimo T, Takizawa A, et al. (2013) Generation of leptin deficient Lepmkyo/Lepmkyo rats and identification of leptin responsive genes in the liver. Physiol Genomics. doi:10.1152/physiolgenomics.00040.2013. 23800849

[60] Kuramoto T, Kuwamura M, Tagami F, Mashimo T, Nose M, et al (2011) Kyoto rhino rats derived by ENU mutagenesis undergo congenital hair loss and exhibit focal glomerulosclerosis. Exp Anim Jpn Assoc Lab Anim Sci 60: 57–63. 10.1538/expanim.60.57 21325752

[61] Mashimo T, Ohmori I, Ouchida M, Ohno Y, Tsurumi T, et al (2010) A missense mutation of the gene encoding voltage-dependent sodium channel (Nav1.1) confers susceptibility to febrile seizures in rats. J Neurosci Off J Soc Neurosci 30: 5744–5753 10.1523/JNEUROSCI.3360-09.2010 PMC6632336 20410126

[62] Quwailid MM, Hugill A, Dear N, Vizor L, Wells S, et al (2004) A gene-driven ENU-based approach to generating an allelic series in any gene. Mamm Genome Off J Int Mamm Genome Soc 15: 585–591 10.1007/s00335-004-2379-z 15457338

[63] Acevedo-Arozena A, Kalmar B, Essa S, Ricketts T, Joyce P, et al (2011) A comprehensive assessment of the SOD1G93A low-copy transgenic mouse, which models human amyotrophic lateral sclerosis. Dis Model Mech 4: 686–700 10.1242/dmm.007237 21540242 PMC3180233

[64] Rogers DC, Fisher EM, Brown SD, Peters J, Hunter AJ, et al (1997) Behavioral and functional analysis of mouse phenotype: SHIRPA, a proposed protocol for comprehensive phenotype assessment. Mamm Genome Off J Int Mamm Genome Soc 8: 711–713. 10.1007/s003359900551 9321461

[65] Malik B, Nirmalananthan N, Bilsland LG, La Spada AR, Hanna MG, et al (2011) Absence of disturbed axonal transport in spinal and bulbar muscular atrophy. Hum Mol Genet 20: 1776–1786 10.1093/hmg/ddr061 21317158 PMC3071673

[66] Kalmar B, Novoselov S, Gray A, Cheetham ME, Margulis B, et al (2008) Late stage treatment with arimoclomol delays disease progression and prevents protein aggregation in the SOD1 mouse model of ALS. J Neurochem 107: 339–350 10.1111/j.1471-4159.2008.05595.x 18673445

[67] Sharp PS, Dick JRT, Greensmith L (2005) The effect of peripheral nerve injury on disease progression in the SOD1(G93A) mouse model of amyotrophic lateral sclerosis. Neuroscience 130: 897–910 10.1016/j.neuroscience.2004.09.069 15652988

[68] Lu C-H, Petzold A, Kalmar B, Dick J, Malaspina A, et al (2012) Plasma neurofilament heavy chain levels correlate to markers of late stage disease progression and treatment response in SOD1(G93A) mice that model ALS. PloS One 7: e40998 10.1371/journal.pone.0040998 22815892 PMC3397981

[69] McHanwell S, Biscoe TJ (1981) The localization of motoneurons supplying the hindlimb muscles of the mouse. Philos Trans R Soc Lond B Biol Sci 293: 477–508. 6115428 10.1098/rstb.1981.0082

[70] Han J-H, Yu T-H, Ryu H-H, Jun M-H, Ban B-K, et al. (2013) ALS/FTLD-linked TDP-43 regulates neurite morphology and cell survival in differentiated neurons. Exp Cell Res. doi:10.1016/j.yexcr.2013.05.025. 23742895

[71] Lu Y, Ferris J, Gao F-B (2009) Frontotemporal dementia and amyotrophic lateral sclerosis-associated disease protein TDP-43 promotes dendritic branching. Mol Brain 2: 30 10.1186/1756-6606-2-30 19781077 PMC2762964

[72] Bittner RE, Anderson LV, Burkhardt E, Bashir R, Vafiadaki E, et al (1999) Dysferlin deletion in SJL mice (SJL-Dysf) defines a natural model for limb girdle muscular dystrophy 2B. Nat Genet 23: 141–142 10.1038/13770 10508505

[73] Stack EC, Dedeoglu A, Smith KM, Cormier K, Kubilus JK, et al (2007) Neuroprotective effects of synaptic modulation in Huntington’s disease R6/2 mice. J Neurosci Off J Soc Neurosci 27: 12908–12915 10.1523/JNEUROSCI.4318-07.2007 PMC6673303 18032664

[74] Irwin S (1968) Comprehensive observational assessment: Ia. A systematic, quantitative procedure for assessing the behavioral and physiologic state of the mouse. Psychopharmacologia 13: 222–257. 5679627 10.1007/BF00401402

[75] Messaoudi M, Rozan P, Nejdi A, Hidalgo S, Desor D (2005) Behavioural and cognitive effects of oligofructose-enriched inulin in rats. Br J Nutr 93 Suppl 1S27–30. 15877891 10.1079/bjn20041348

[76] Duysen EG, Li B, Darvesh S, Lockridge O (2007) Sensitivity of butyrylcholinesterase knockout mice to (–)-huperzine A and donepezil suggests humans with butyrylcholinesterase deficiency may not tolerate these Alzheimer’s disease drugs and indicates butyrylcholinesterase function in neurotransmission. Toxicology 233: 60–69 10.1016/j.tox.2006.11.069 17194517

[77] Ingman K, Sallinen J, Honkanen A, Korpi ER (2004) Comparison of deramciclane to benzodiazepine agonists in behavioural activity of mice and in alcohol drinking of alcohol-preferring rats. Pharmacol Biochem Behav 77: 847–854 10.1016/j.pbb.2004.02.015 15099931

