# Supplementary figures and images for: A Nonsense Mutation in Mouse Tardbp Affects TDP43 Alternative Splicing Activity and Causes Limb-Clasping and Body Tone Defects
Source: PLoS One. 2014 Jan 21;9(1):e85962. doi: 10.1371/journal.pone.0085962 (PMC3897576; doi:10.1371/journal.pone.0085962)

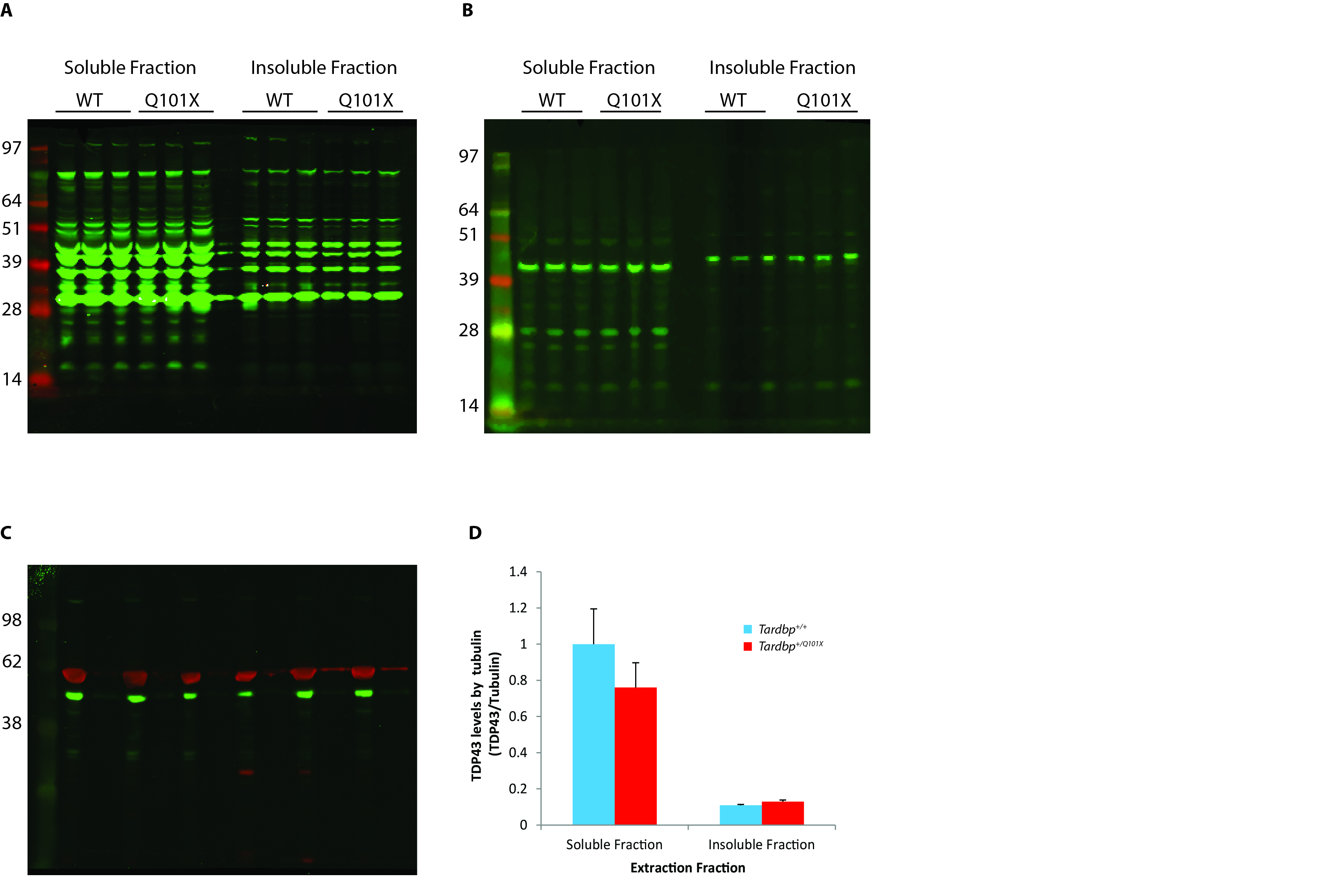

Supplement: Figure S1 — TDP43 protein levels do not change in Tardbp+/Q101X mice. (A, B) N-terminal anti TDP-43 antibodies do not show any novel truncated TDP-43 band in Tardbp+/Q101X soluble or RIPA-insoluble brain fractions from 18 month-old males. N-terminal antibodies used were: (A) Cosmo Bio (CAC-TIP-TD-P07) and (B) Abcam (ab50930). (C) No significant differences in full-length TDP43 protein levels relative to tubulin between Tardbp+/+ (0.99±0.20) and Tardbp+/Q101X (0.76±0.14) using an antibody directed against C-terminus of TDP43 (Proteintech 12892-1-AP). TDP43 levels (green) were assessed from RIPA soluble (p = 0.375) and RIPA-insoluble fractions (p = 0.123) from whole spinal cord lysates using 3 mice per genotype at 18 months of age. Tubulin (red) was used as a loading control. Data are mean±SEM. (TIF) [file pone.0085962.s001.tif]

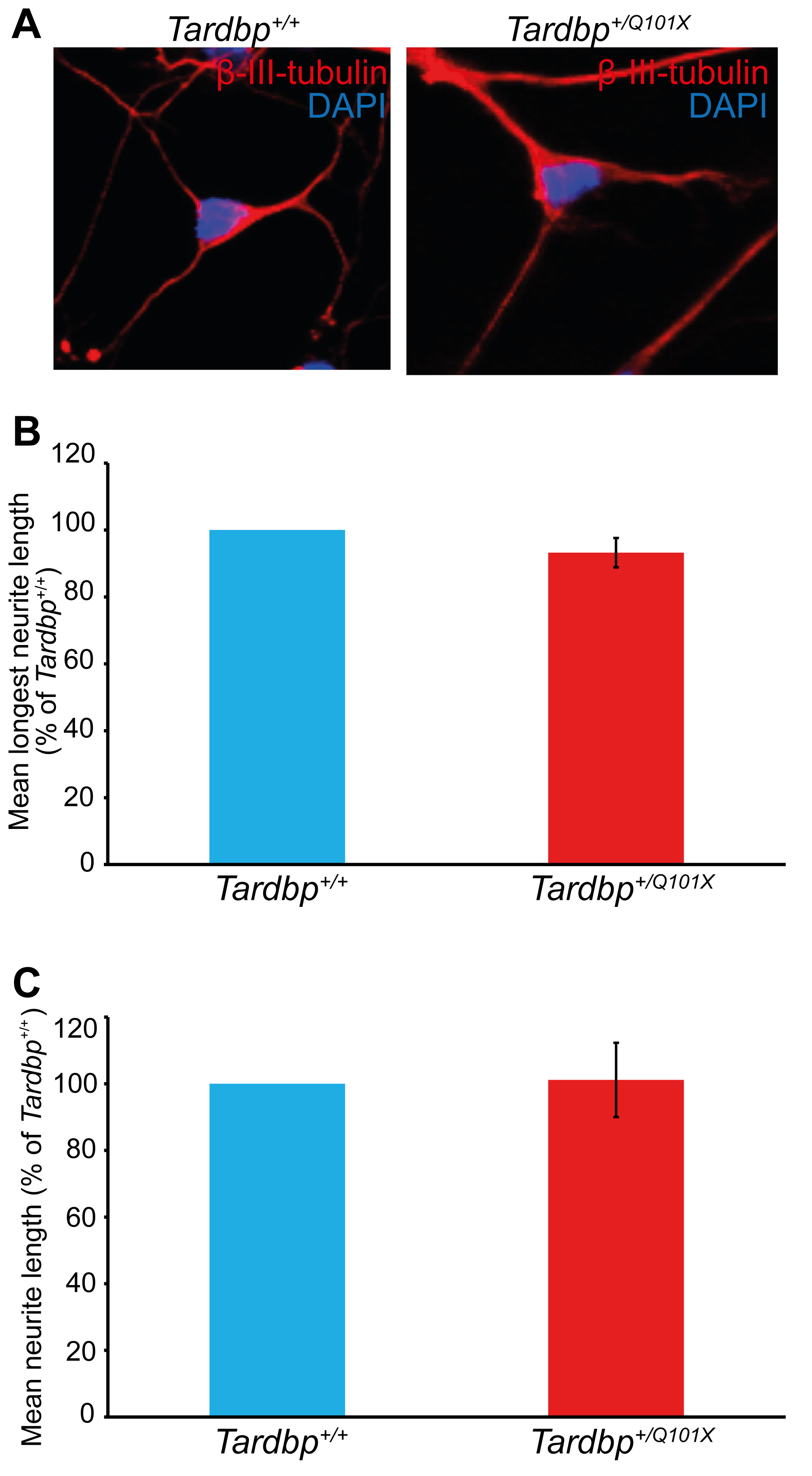

Supplement: Figure S2 — Neurite outgrowth of primary embryonic motor neurons is not affected by the Tardbp Q101X mutation. (A) Representative images of primary embryonic motor neurons stained for the neuronal marker B-III tubulin (red) and DAPI (blue) from Tardbp+/+ and Tardbp+/Q101X embryos. (B) Mean longest neurite length was not significantly different between Tardbp+/+ and Tardbp+/Q101X motor neurons (n = 175 and n = 117 neurons, respectively). (C) Neither were any differences seen between mean neurite length of Tardbp+/+ and Tardbp+/Q101X motor neurons. Data are mean±SEM from 3 independent experiments. (TIF) [file pone.0085962.s002.tif]

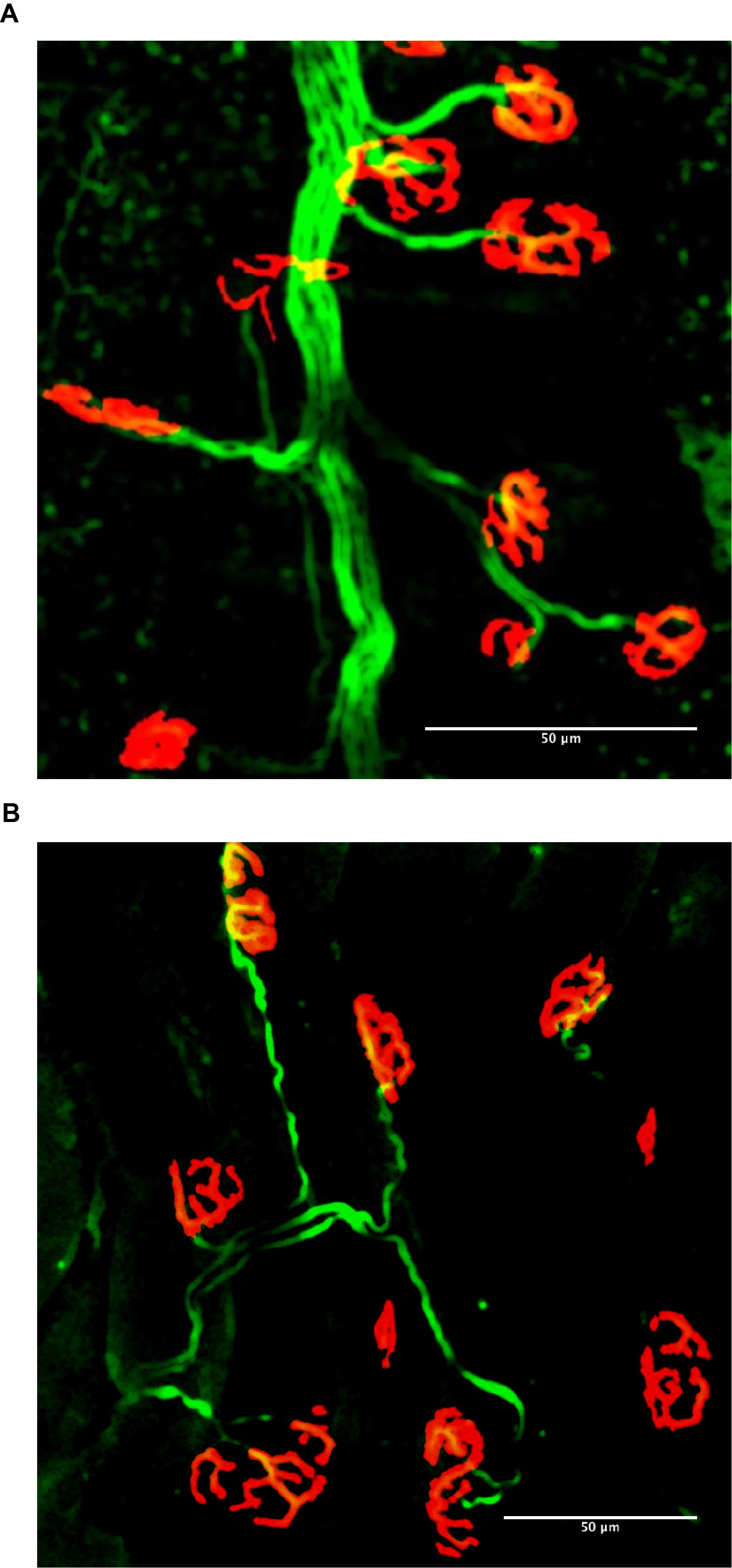

Supplement: Figure S3 — Tardbp Q101X mice present a normal innervation pattern of the external abdominal oblique muscle. (A, B) Representative immunofluorescence images of whole mount abdominal oblique muscle from Tardbp+/+ (A) and Tardbp+/Q101X (B) mice at ∼1 year of age. A normal innervation pattern was present for both genotypes. Postsynaptic, presynaptic and axonal regions were identified by acetylcholine receptor (red), synaptic vesicle protein (green) and neurofilament (green) staining, respectively. Scale bar represent 50 µm in both images. (TIF) [file pone.0085962.s003.tif]

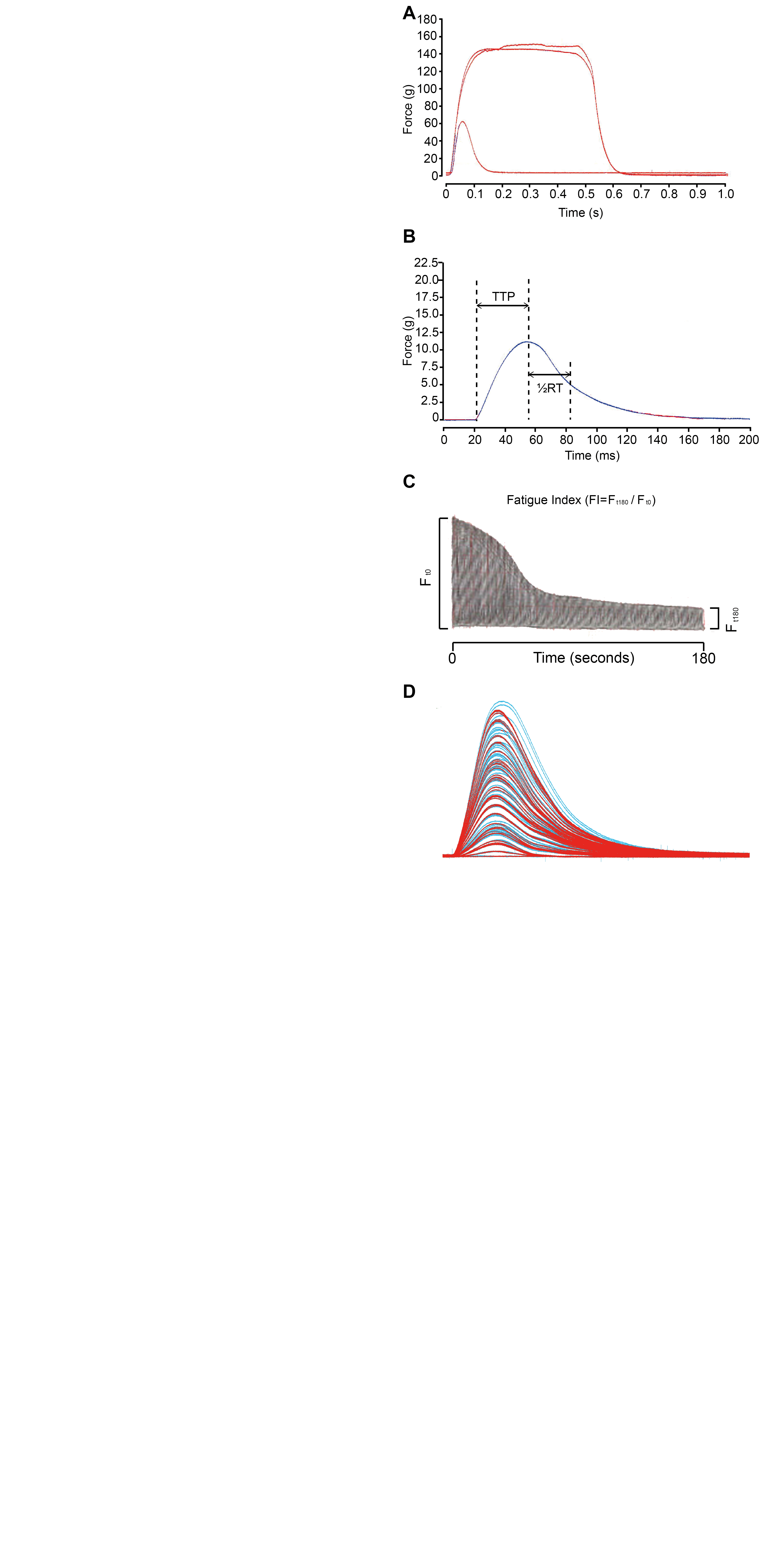

Supplement: Figure S4 — Examples of recordings from in vivo assessment of neuromuscular function of male Tardbp+/+ mice at 18 months of age. (A) Example recording of maximum twitch (smaller peak) and tetanic force (larger peak) from TA muscle. (B) Example trace from an EDL muscle illustrating how contraction time (TTP) and relaxation time (½RT) are calculated from maximum twitch force recordings. (C) Example trace demonstrating motor unit number estimation of the EDL. (D) Trace recording of fatigue characteristic of an EDL muscle, where the fatigue index (FI) is calculated as the ratio of force after 180 seconds (F180) compared to initial force (F0). (TIF) [file pone.0085962.s004.tif]

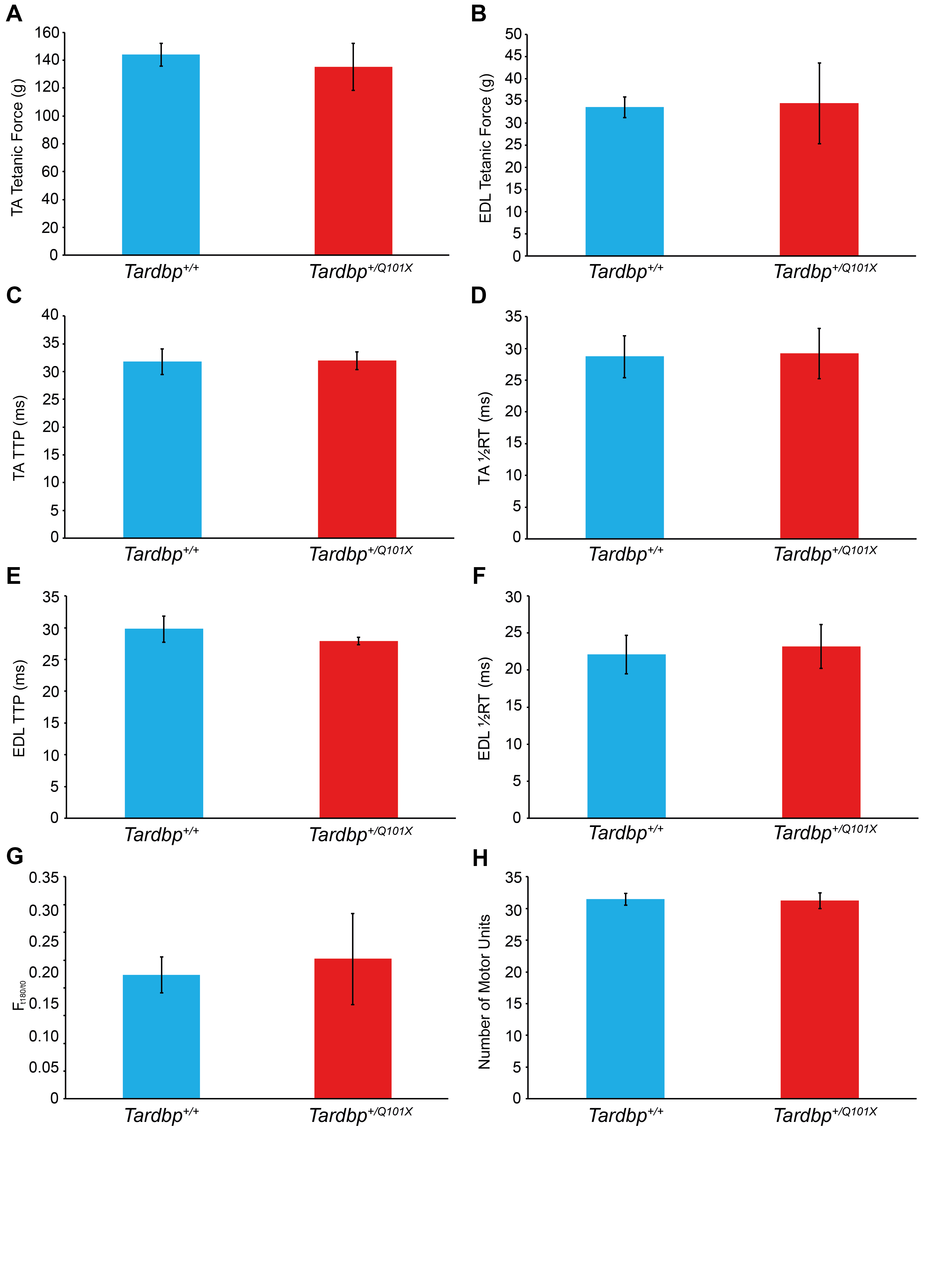

Supplement: Figure S5 — No evidence of neuromuscular dysfunction in male Tardbp +/Q101X mice at 18 months of age. (A) Maximum tetanic force recorded from TA muscles was not significantly different between Tardbp+/+ (n = 12 muscles) and Tardbp+/Q101X (n = 6) mice. (B) No difference was seen in the maximum tetanic force of EDL muscles in Tardbp+/+ (n = 8) and Tardbp+/Q101X (n = 5) mice. (C&D) Assessment of TA contraction (TTP) and relaxation (½RT) characteristics in Tardbp+/+ and Tardbp+/Q101X mice (n = 11, and n = 6, respectively) did not reveal any significant differences. (E&F) EDL muscle contraction (TTP) and relaxation (½RT) characteristics did not differ between Tardbp+/+ and Tardbp+/Q101X mice (n = 8 and n = 5, respectively) (G) A fatigue index of EDL muscles was established but did not show any difference between Tardbp+/+ (n = 7) and Tardbp+/Q101X (n = 3) mice. (H) The number of motor units innervating EDL muscles was assessed but also failed to show any difference between Tardbp+/+ (n = 8) and Tardbp+/Q101X (n = 4) mice. (TIF) [file pone.0085962.s005.tif]

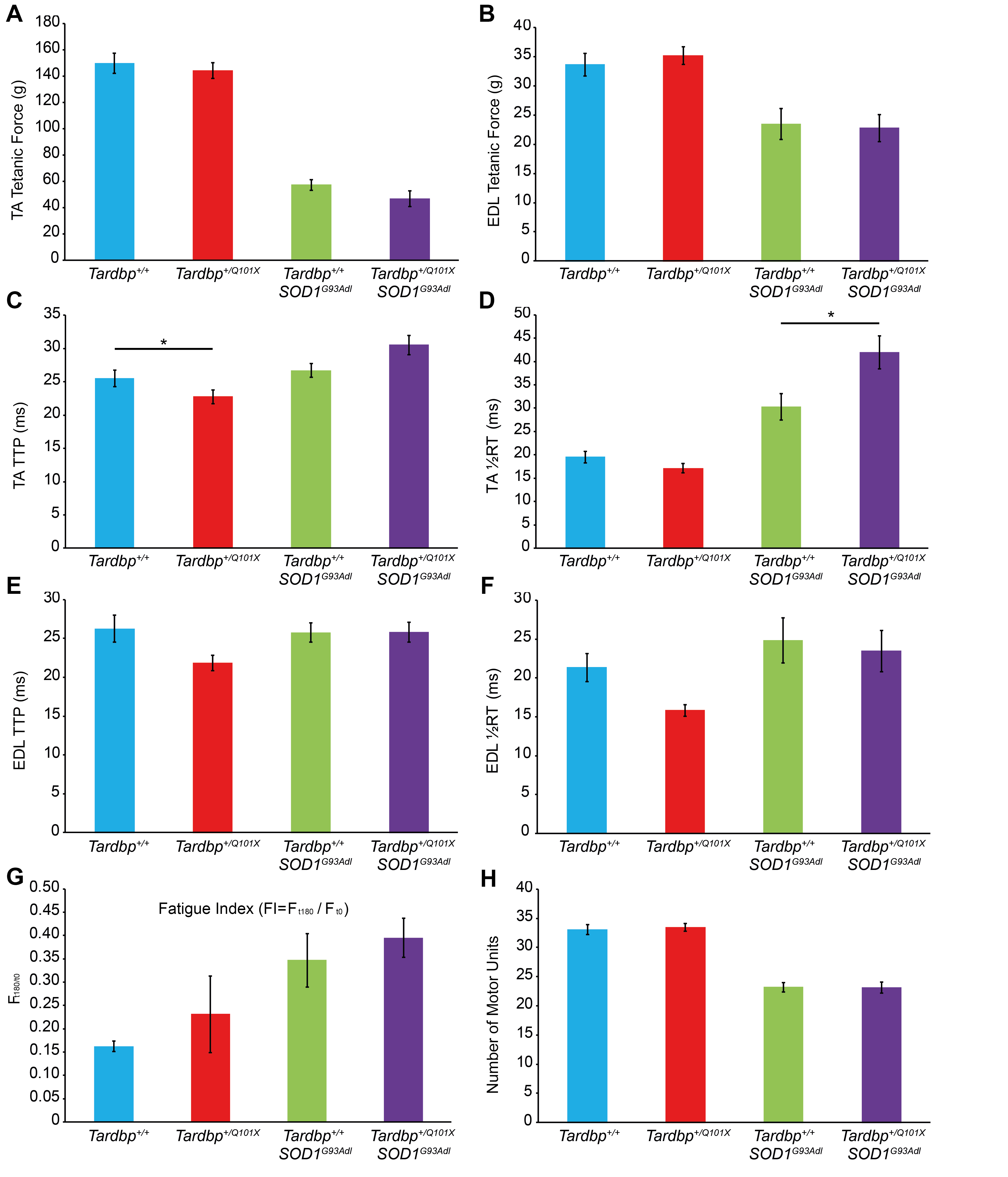

Supplement: Figure S6 — Assessment of hindlimb neuromuscular function in male Tardbp+/Q101X , SOD1G93Adl mice. (A) Maximum tetanic force recorded from the TA was not significantly different between Tardbp+/+ and Tardbp+/Q101X mice (both n = 10), or between Tardbp+/+, SOD1G93Adl and Tardbp+/Q101X, SOD1G93Adl mice (both n = 10). (B) EDL tetanic force did not differ between Tardbp+/+ (n = 8) and Tardbp+/Q101X (n = 9) mice, or between Tardbp+/+, SOD1G93Adl (n = 10) and Tardbp+/Q101X, SOD1G93Adl (n = 9) mice. (C&D) Contraction (TTP) and relaxation (½RT) of TA muscles did not differ between Tardbp+/+ (n = 9 and n = 8) and Tardbp+/Q101X (n = 13, n = 12) mice, and although TTP did not differ between Tardbp+/+, SOD1G93Adl (n = 9) and Tardbp+/Q101X, SOD1G93Adl (n = 10) mice, relaxation time was significantly slower in Tardbp+/Q101X, SOD1G93Adl mice compared to Tardbp+/+, SOD1G93Adl mice (both n = 10; p = 0.007). (E) Contraction of EDL muscles was not different between Tardbp+/+ (n = 8) and Tardbp+/Q101X mice (n = 9) or between Tardbp+/+, SOD1G93Adl (n = 10) and Tardbp+/Q101X, SOD1G93Adl (n = 10) mice. (F) EDL relaxation time was significantly quicker in Tardbp+/Q101X (n = 9) mice compared to Tardbp+/+ mice (n = 7; p<0.05), however no difference was observed between Tardbp+/+, SOD1G93Adl (n = 10) and Tardbp+/Q101X, SOD1G93Adl (n = 10) mice. (G) Fatigue characteristics of the EDL, defined as the FI, did not differ between Tardbp+/+ (n = 9) and Tardbp+/Q101X (n = 8) mice, or between Tardbp+/+, SOD1G93Adl (n = 7) and Tardbp+/Q101X, SOD1G93Adl (n = 7) mice. (H) The number of surviving motor units of EDL muscles was not significantly different between Tardbp+/+ (n = 8) and Tardbp+/Q101X (n = 8) mice, or between Tardbp+/+, SOD1G93Adl (n = 8) and Tardbp+/Q101X, SOD1G93Adl (n = 10) mice. (TIF) [file pone.0085962.s006.tif]

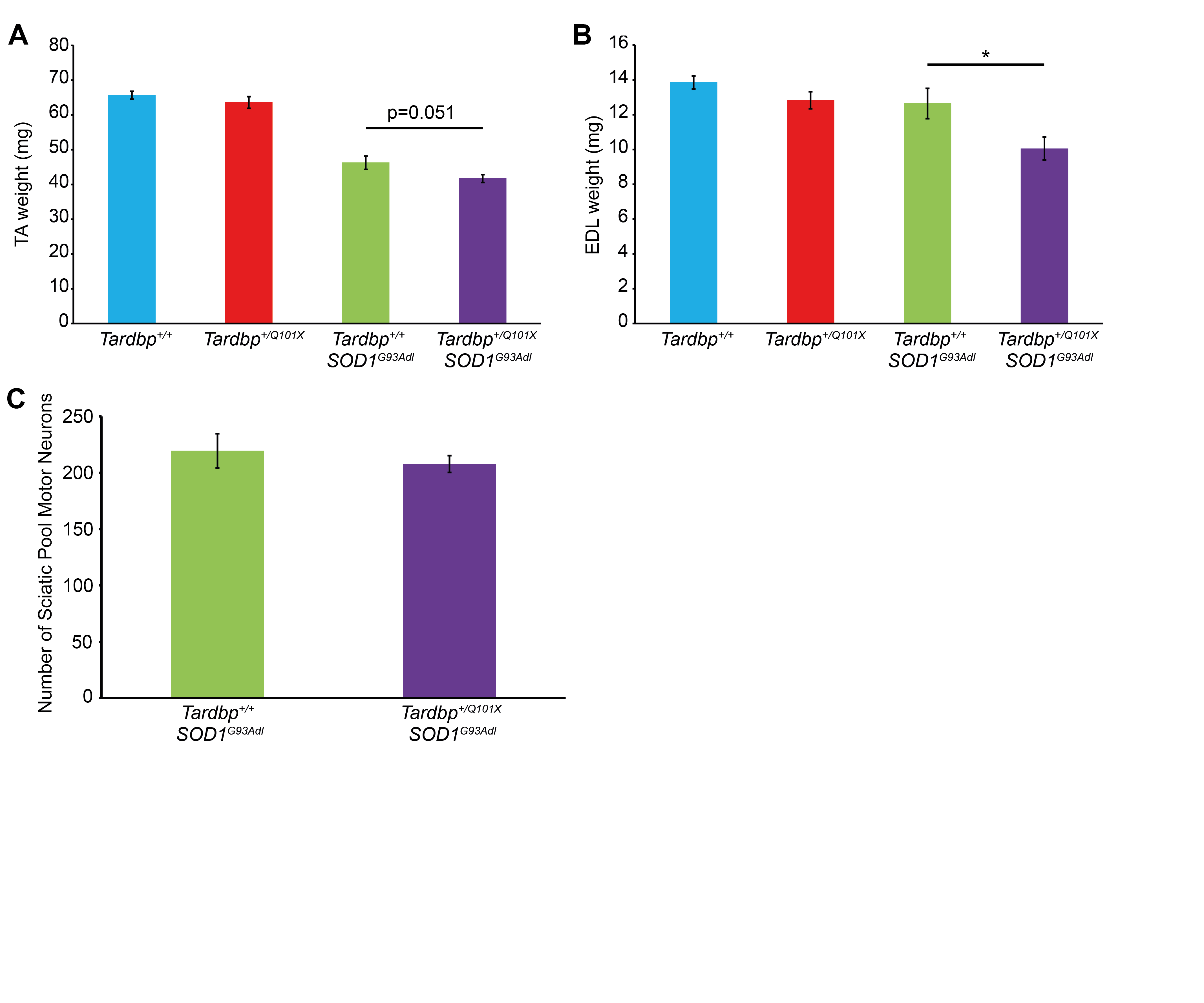

Supplement: Figure S7 — Muscle weights and motor neuron survival in male Tardbp+/+ , SOD1G93Adl and Tardbp+/Q101X , SOD1G93Adl mice at 32–33 weeks of age. (A) No difference between TA muscle weights of Tardbp+/+ (n = 12) and Tardbp+/Q101X (n = 17) mice, or between Tardbp+/+, SOD1G93Adl (n = 9) and Tardbp+/Q101X, SOD1G93Adl (n = 12) mice (p = 0.051). (B) EDL muscle weight was similar between Tardbp+/+ (n = 12) and Tardbp+/Q101X (n = 17) mice, but was showed a significant difference between Tardbp+/+, SOD1G93Adl (n = 8) and Tardbp+/Q101X, SOD1G93Adl (n = 8) mice (p = 0.035). (C) The number of motor neurons of the sciatic pool (L2-L6) was counted in Tardbp+/+, SOD1G93Adl (n = 3) and Tardbp+/Q101X, SOD1G93Adl (n = 4) mice and did not reveal any differences. (TIF) [file pone.0085962.s007.tif]
