# Supplementary material for: A Nonsense Mutation in Mouse Tardbp Affects TDP43 Alternative Splicing Activity and Causes Limb-Clasping and Body Tone Defects
Source: PLoS One. 2014 Jan 21;9(1):e85962. doi: 10.1371/journal.pone.0085962 (PMC3897576; doi:10.1371/journal.pone.0085962)
Supplement: Table S1 — Normal hindlimb neuromuscular function in Tardbp+/Q101X male mice at 18 months of age. (DOCX) [file pone.0085962.s008.docx]

**Table S1: Normal hindlimb neuromuscular function in *Tardbp^+/Q101X^* male mice at 18 months of age**

|  | **Genotype** | |
| --- | --- | --- |
|  | ***Tardbp^+/+^*** | ***Tardbp^+/Q101X^*** |
| **Tibialis anterior** | | |
| Twitch Force (g) | 47.9±2.1 (n=12) | 52.6g±4.5g (n=6) |
| Tetanic Force (g) | 144.1g±8.1g (n=10) | 135.3g±16.8g (n=6) |
| TTP (ms) | 31.8ms±2.3ms (n=11) | 32.0ms±1.6ms (n=6) |
| ½RT (ms) | 28.7ms±3.3ms (n=11) | 29.2ms±3.9ms, (n=6) |
| TA Weight (mg) | 59.6mg±3.1mg (n=12) | 62.4mg±6.5mg (n=4) |
| **Extensor digitorum longus** | | |
| Twitch Force (g) | 12.2g±0.3g (n=8) | 13.8g±4.0g (n=5) |
| Tetanic Force (g) | 33.6g±2.6g (n=8) | 34.5g±9.1g (n=5) |
| TTP (ms) | 29.8ms±2.0 (n=8) | 27.9ms±0.6ms (n=5) |
| ½RT (ms) | 22.13ms±2.60ms (n=8) | 23.2ms±3.0ms (n=5) |
| FI | 0.22±0.03 (n=7) | 0.25±0.08 (n=3) |
| Number of surviving motor units | 31.5±0.8 (n=8) | 31.3±1.3 (n=4) |
| EDL Weight (mg) | 13.2mg±0.9mg (n=11) | 16.3mg±2.7mg (n=4) |
