# Supplementary material for: A Nonsense Mutation in Mouse Tardbp Affects TDP43 Alternative Splicing Activity and Causes Limb-Clasping and Body Tone Defects
Source: PLoS One. 2014 Jan 21;9(1):e85962. doi: 10.1371/journal.pone.0085962 (PMC3897576; doi:10.1371/journal.pone.0085962)
Supplement: Table S2 — The Q101X mutation in TDP43 does not affect neuromuscular function in 32–33 week old SOD1G93Adl mice. (DOCX) [file pone.0085962.s009.docx]

**Table S2: The Q101X mutation in TDP43 does not affect neuromuscular function in 32-33 week old *SOD1^G93Adl^* mice**

|  | Genotype | | | |
| --- | --- | --- | --- | --- |
|  | *Tardbp^+/+^* | *Tardbp^+/Q101X^* | *Tardbp^+/+^*, *SOD1^G93Adl^* | *Tardbp^+/Q101X^*, *SOD1^G93Adl^* |
| **TA** | | | | |
| Twitch Force (g) | 40.9g±4.5g (n=13) | 46.1g±2.0g (n=13) | 21.0g±1.6g (n=10) | 19.6g±2.7g (n=10) |
| Tetanic Force (g) | 150.9g±7.8g (n=10) | 144.4g±5.9g (n=10) | 57.3g±4.1g (n=10) | 46.7g±6.0g (n=10) |
| TTP (ms) | 25.3ms±1.3ms (n=9) | 22.8ms±1.0ms (n=13) | 26.7ms±1.0ms (n=9) | 30.6ms±1.5ms (n=10) |
| ½RT (ms) | 19.6ms±1.2ms (n=8) | 17.2ms±1.0ms (n=12) | 30.4ms±2.8ms (n=10) | 42.0ms±3.5ms (n=10) |
| TA Weight (mg) | 65.7mg±1.2mg (n=12) | 63.5mg±1.7mg (n=17) | 46.3mg±1.9mg (n=9) | 41.7mg±1.2mg (n=12) |
| **EDL** | | | | |
| Twitch Force (g) | 12.0g±0.5g (n=8) | 10.5g±1.1g (n=9) | 7.5g±0.8 (n=9) | 7.3g±0.8g (n=9) |
| Tetanic Force (g) | 33.7g±1.9g (n=8) | 35.3g±4.5g (n=9) | 23.5g±2.6g (n=10) | 22.8g±2.3g (n=9) |
| TTP (ms) | 26.3ms±1.8ms (n=8) | 21.9ms±1.0 (n=9) | 25.8ms±1.2ms (n=10) | 25.9ms±1.3ms (n=10) |
| ½RT (ms) | 21.4ms±1.8ms (n=7) | 15.9ms±0.7ms (n=9) | 24.9ms±3.0ms (n=10) | 23.6ms±2.6ms (n=10) |
| FI | 0.16±0.01 (n=9) | 0.23±0.8 (n=8) | 0.34±0.05 (n=7) | 0.39±0.04 (n=7) |
| Number of surviving motor units | 33.1±0.89 (n=7) | 33.5±0.6 (n=8) | 23.2±0.8 (n=8) | 23.2±0.9 (n=10) |
| EDL Weight (mg) | 13.9mg±0.4mg (n=12) | 12.8mg±0.5mg (n=17) | 12.7mg±0.9mg (n=8) | 10.1mg±0.7mg (n=8) |
